# Muscle fiber type-dependence effect of exercise on genomic networks in aged mice models

**DOI:** 10.18632/aging.204024

**Published:** 2022-04-19

**Authors:** Sun Min Lee, Min Chul Lee, Woo Ri Bae, Kyung Jin Yoon, Hyo Youl Moon

**Affiliations:** 1 Department of Physical Education, Seoul National University, Seoul, South Korea; 2 Institute of Sport Science, Seoul National University, Seoul, South Korea; 3 Institute on Aging, Seoul National University, Seoul, South Korea; 4 Department of Sports Medicine, College of Health Science, CHA University, Pocheon, South Korea

**Keywords:** muscle fiber type, exercise, aging, apoptosis, myogenesis

## Abstract

Skeletal muscles are made up of various muscle fiber type including slow and fast-twitch fibers. Because each muscle fiber has its own physiological characteristics, the effects of aging and exercise vary depending on the type of muscle fiber. We used bioinformatics screening techniques such as differentially expressed gene analysis, gene ontology analysis and gene set enrichment analysis, to try to understand the genetic differences between muscle fiber types. The experiment and gene expression profiling in this study used the soleus (SOL, slow-twitch muscle) and gastrocnemius (GAS, fast-twitch muscle). According to our findings, fatty acid metabolism is significantly up-regulated in SOL compared to GAS, whereas the glucose metabolism pathway is significantly down-regulated in SOL compared to GAS.

Furthermore, apoptosis and myogenesis patterns differ between SOL and GAS. SOL did not show differences in apoptosis due to the aging effect, but apoptosis in GAS was significantly up-regulated with age. Apoptosis in GAS of old groups is significantly reduced after 4 weeks of aerobic exercise, but no such finding was found in SOL. In terms of myogenesis, exercise intervention up-regulated this process in GAS of old groups but not in SOL. Taken together, muscle fiber type significantly interacts with aging and exercise. Despite the importance of the interaction between these factors, large-scale gene expression data has rarely been studied. We hope to contribute to a better understanding of the relationship between muscle fiber type, aging and exercise at the molecular level.

## INTRODUCTION

Type I (slow-twitch) and type II (fast-twitch) muscle fibers make up the mammalian skeletal muscle, which is a very heterogeneous tissue [1]. Different factors, such as aging, exercise, and diseases, affect type I and type II fiber differently. Previous studies have shown that aging has a different impact depending on the muscle fiber type composition. Type II fibers significantly decreased with age, whereas type I fibers were unaffected [2–4]. Furthermore, glycolysis and glycogen metabolism were also up-regulated in type I fibers as they aged, whereas they were down-regulated in type II fibers [2]. As a result, the interaction between aging and muscle fiber type is important in terms of muscle atrophy and metabolism, though the exact cellular mechanism is still unknown.

One of the most effective strategies for rejuvenation, cognitive function, and disease prevention, such as metabolic syndrome, is exercise [5–7]. Exercise has different effects depending on muscle fiber type, in addition to aging. Acute exercise significantly increased the expression of lipolytic complex genes (*ATGL, CGI-58, G0S2*) in the soleus (SOL) and red section of the gastrocnemius (GAS), whereas it decreased the expression of lipolytic complex genes (*ATGL, CGI-58, G0S2*) in the white section of the gastrocnemius (GAS) [8]. The effect of exercise on muscle hypertrophy varies depending on the muscle fiber type. Resistance training improves the muscle *mass*-to-body ratio in fast-twitch muscles but not in slow-twitch muscles [9]. Moreover, exercise causes changes in the composition of muscle fiber types. Type IIA fibers were found to be increased in previous studies, while type I fibers remained unchanged or decreased [10–12]. Endurance training, on the other hand, can cause a shift toward type I fibers [13, 14]. To summarize, exercise has a close relationship with muscle fiber type, as evidenced by numerous studies, and the relationship between exercise and muscle fiber type should be investigated to better understand the numerous benefits of exercise.

Among skeletal muscles, SOL and GAS have a different muscle fiber type composition than the rest of the skeletal muscles. In mouse SOL contains a high percentage of slow twitch fibers (type I 37.42 ± 8.20%, IIA 38.62 ± 6.81%, IIAD 18.74 ± 6.95% and IID 5.69 ± 3.09%), whereas GAS is primarily made up of fast twitch fibers (type IIB 54.42 ± 8.11%, IIDB 19.37 ± 2.98%, IID 2.26 ± 2.24%, IIAD 12.40 ± 2.34%, IIA 5.73 ± 3.24% and I 5.74 ± 2.55%) [15]. Because SOL and GAS have remarkably different muscle fiber type composition, SOL and GAS exhibit different patterns with aging. In rats, SOL was not significantly changed in muscle weights by aging, while GAS was decreased [16]. In humans, SOL thickness did not differ in older vs. younger groups, but GAS atrophied in older vs. younger groups [17]. SOL has the characteristics of a slow-twitch muscle, whereas GAS has the characteristics of a fast-twitch muscle, due to the fact that fast-twitch fiber tends to atrophy more with age than slow-twitch fiber.

In addition to the effect of aging, exercise has different effects on SOL and GAS. SOL has more DEGs induced by exercise than GAS in terms of number. The stress-response gene activating transcription factor 3 (*Atf3*) [18], was only up-regulated in SOL and not in GAS [19]. Although strenuous exercise causes an increase in apoptosis in both SOL and GAS, the patterns of apoptosis are different (intrinsic vs. extrinsic pathway) [20]. Furthermore, the effects of exercise on brain-derived neurotrophic factor (BDNF, known to promote neuron survival) and sequestosome 1 (SQSTM1, a receptor that accumulates when autophagy is inhibited) differ between SOL and GAS [21]. Short-term exercise increased *BDNF* expression in SOL but not in GAS (for 5 days). *SQSTM1* gene expression was increased in SOL, but not in GAS, despite chronic exercise (for 8 weeks) [22]. In summary, exercise has a different effect on between SOL and GAS at the molecular level.

Taken together, muscle fiber type, aging and exercise relevantly interact with each other. Because the effects of aging and exercise differ significantly depending on muscle fiber type, and muscle fiber type composition is linked to diseases such as obesity, diabetes, and cancer [23–26], it’s critical to look into the interaction between muscle fiber type and various factors. Despite these requirements, large-scale gene expression data has rarely been examined in terms of the effects of aging and exercise on different muscle fiber types. As a result, we used bioinformatics tools to perform systematic gene expression profiling in an attempt to elucidate the genetic relationship between muscle fiber type, aging, and exercise.

## RESULTS

### Principal component analysis (PCA) plots and heatmaps

**Figure 1:**
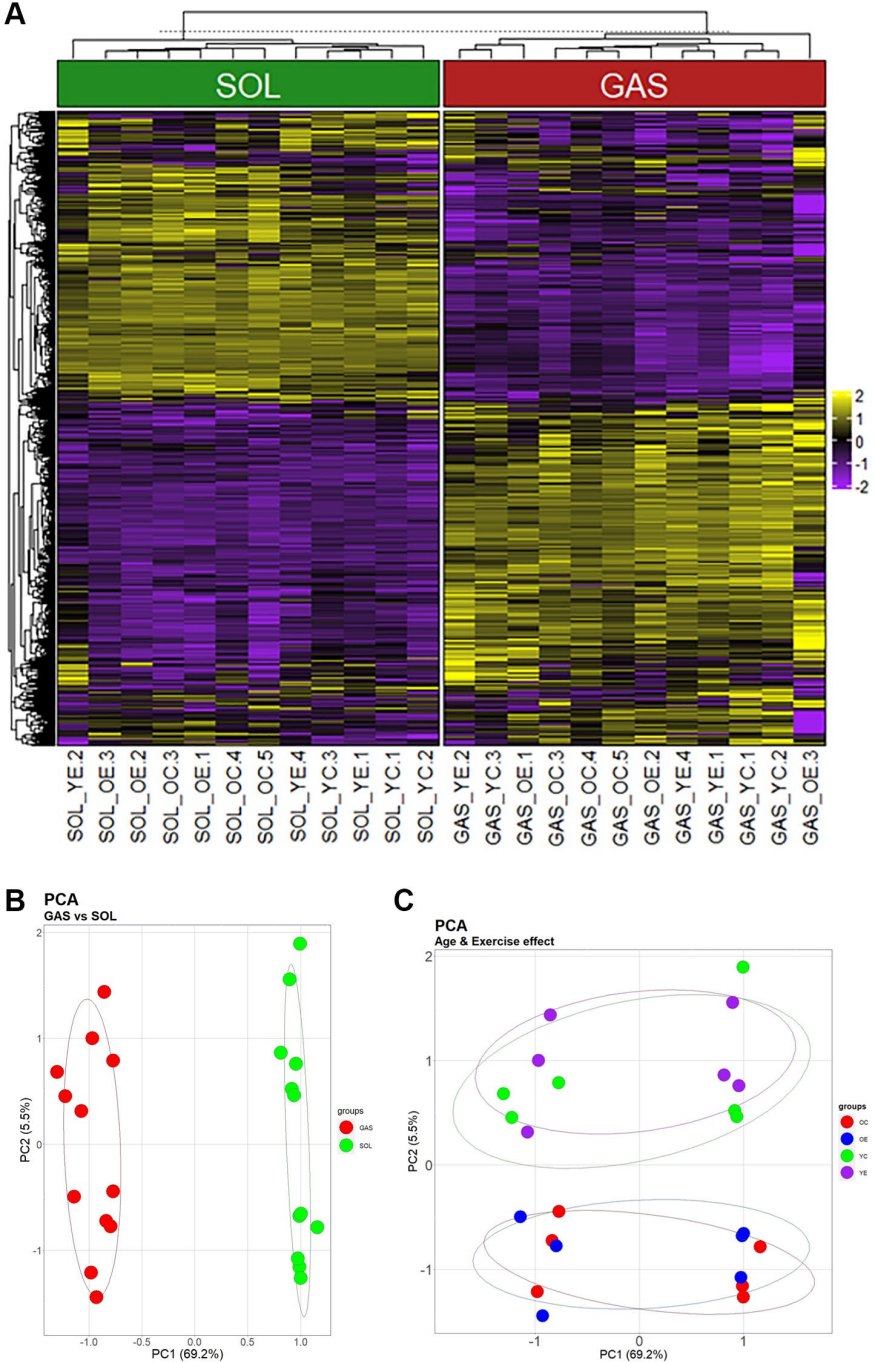
**Heatmap and PCA showing the effects of muscle fiber type, aging, and exercise on gene expression.** (**A**) Heatmap using the 1268 genes that passed screening. (i) Values for genes with median value of <5 and (ii) SD of bottom 25% were excluded. Yellow indicates high expression, and blue indicates low expression. (**B**) and (**C**) are the same plot, but they were clustered by another PC. (**B**) Clusters separated by PC1 (69.2% of total variance) (**C**) Groups divided by PC2 (5.5% of total variance).

To understand the expression profiles of the identified genes, PCA plots and heatmaps are presented. SOL and GAS were divided into two clusters, as illustrated by the dendrogram (Figure 1A). DEGs were mostly obtained in the comparison between types of muscle fibers (SOL vs. GAS) (Figure 1A and Supplementary Tables 1, 2). PCA plots also indicated that two clusters (SOL vs. GAS) were clearly separated by principal component 1 (PC1, 69.2%) (Figure 1B). Considerable separation between the young and old groups was achieved by principal component 2 (PC2, 5.5%) (Figure 1C).

### Identification of DEGs between YC vs. OC in SOL and GAS and commonly expressed DEGs

**Figure 2:**
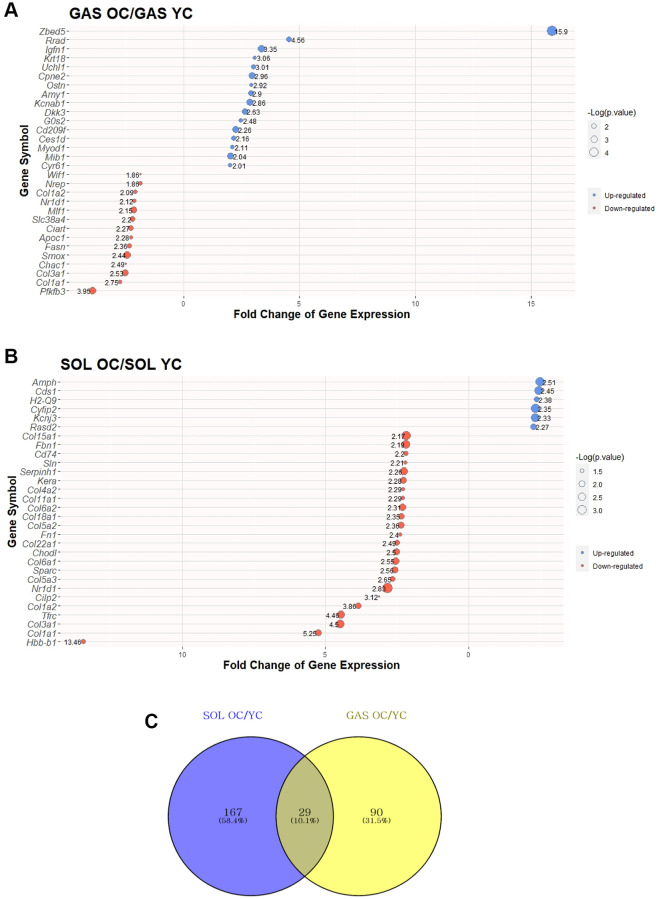
**Top 30 DEGs between “YC vs. OC” in SOL and GAS and Venn diagram to find commonly expressed DEGs.** The point sizes of (**A**) and (**B**) represent ‒log_10_ (*p*-value). For (**A**) and (**B**), blue points indicate upregulated genes, and red points indicate downregulated genes. (**A**) Top 30 DEGs in GAS OC/YC. (**B**) Top 30 DEGs in SOL OC/YC. (**C**) Venn diagram of DEGs between YC and OC in SOL and GAS. For commonly expressed genes, detailed fold changes and *p*-values are shown in the Table 1.

To investigate the effect of aging on SOL and GAS, DEG analysis was performed. DEGs were determined following >1.5-fold change in random expectation (*p* < 0.05). A total of 286 DEGs were obtained with aging in both SOL and GAS, including 196 DEGs in SOL OC/YC and 119 DEGs in GAS OC/YC (Supplementary Table 1). Of the 196 DEGs in SOL OC/YC, 90 genes were upregulated, and 106 were downregulated (Supplementary Table 1). However, among 119 DEGs in GAS OC/YC, 71 genes were upregulated, and 48 were downregulated (Supplementary Table 1). For each of SOL and GAS, the 30 DEGs with the largest absolute value of fold change are plotted (Figure 2A, 2B).

To determine how many of the genes were changed owing to aging in both SOL and GAS, we identified 29 commonly expressed DEGs (Figure 2C and Table 1). Of the 29 common DEGs, only cluster of differentiation 74 (*Cd74*) and TYRO protein tyrosine kinase-binding protein (*Tyrobp*) exhibited a different pattern between SOL and GAS (Table 1). For *Cd74* and *Tyrobp*, the fold change of OC/YC showed downregulation in SOL, whereas it showed upregulation in GAS (downregulation in SOL OC/YC and upregulation in GAS OC/YC) (Table 1). Excluding *Cd74* and *Tyrobp*, 27 of 29 overlapped DEGs showed similar patterns between SOL and GAS (Table 1). For seven common DEGs, the fold change of OC/YC exhibited upregulation in both SOL and GAS (upregulated in both SOL OC/YC and GAS OC/YC) (Table 1). In contrast, for 20 of 29 common DEGs, the fold change of OC/YC displayed downregulation in both SOL and GAS (downregulation in both SOL OC/YC and GAS OC/YC) (Table 1).

**Table 1:** The commonly expressed DEGs for aging effect.

**Gene Symbol**	**SOL OC/SOL YC**	***p*.value**	**GAS OC/GAS YC**	***p*.value**
** Up-regulated in both (SOL and GAS)**				
*Amy1*	1.88	0.0042	2.90	0.0109
*Cd209f*	1.53	0.0113	2.26	0.0034
*Rhpn2*	1.67	0.0140	1.73	0.0103
*Cds1*	2.45	0.0023	1.64	0.0027
*Myh14*	1.59	0.0057	1.59	0.0045
*Cidea*	1.57	0.0006	1.51	0.0434
*Htatip2*	1.55	0.0359	1.51	0.0049
** Down-regulated in both (SOL and GAS)**				
*Creb3l1*	−1.59	0.0046	−1.52	0.0309
*Dbp*	−1.66	0.0101	−1.54	0.0094
*Col6a1*	−2.55	0.0071	−1.54	0.0317
*Loxl2*	−2.07	0.0184	−1.56	0.0048
*Meg3*	−2.03	0.0028	−1.58	0.0161
*Rian*	−1.53	0.0076	−1.62	0.0303
*Col5a1*	−2.03	0.0032	−1.65	0.0011
*Eln*	−2.13	0.0023	−1.68	0.0014
*S100a4*	−1.55	0.0422	−1.69	0.0267
*Aplnr*	−1.92	0.0077	−1.71	0.0026
				
*Col15a1*	−2.17	0.0013	−1.71	0.0109
*Col4a2*	−2.29	0.0371	−1.75	0.0000
*Itm2a*	−1.75	0.0031	−1.77	0.0314
*Col5a3*	−2.65	0.0165	−1.81	0.0021
*Col5a2*	−2.36	0.0101	−1.82	0.0063
*Tfrc*	−4.46	0.0038	−1.84	0.0013
*Col1a2*	−3.86	0.0152	−2.09	0.0231
*Nr1d1*	−2.83	0.0004	−2.12	0.0187
*Col3a1*	−4.50	0.0019	−2.53	0.0026
*Col1a1*	−5.25	0.0091	−2.75	0.0204

*Cd74* ^*^	−2.20	0.0253	1.50	0.0216
*Tyrobp* ^*^	−1.75	0.0348	1.56	0.0234

Genes with ^*^ are different pattern between SOL and GAS.

### Identification of DEGs between YC vs. YE in SOL and GAS and commonly expressed DEGs

**Figure 3:**
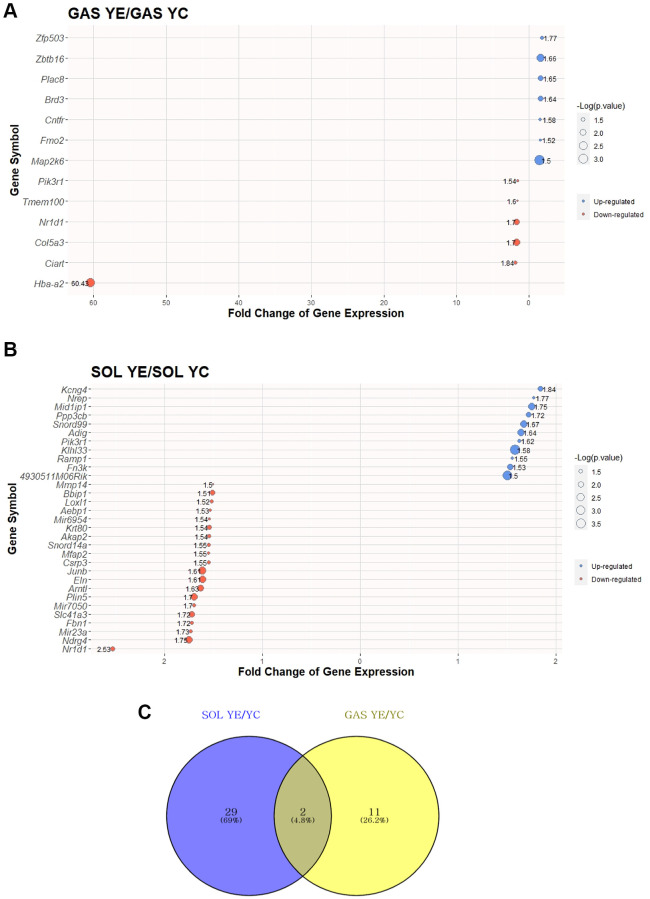
**Top 30 DEGs between “YC vs. YE” in SOL and GAS and Venn diagram to find commonly expressed DEGs.** The point sizes of (**A**) and (**B**) represent ‒log_10_ (*p*-value). For (**A**) and (**B**), blue points indicate upregulated genes, and red points indicate downregulated genes. (**A**) Top 30 DEGs in GAS YE/YC. (**B**) Top 30 DEGs in SOL YE/YC. (**C**) Venn diagram of DEGs between YC and YE in SOL and GAS. For commonly expressed genes, detailed fold changes and *p*-values are shown in the Table 2.

To elucidate the effect of exercise on SOL and GAS in the young groups, DEG analysis was performed. DEGs were identified following >1.5-fold change in random expectation (*p* < 0.05). A total of 42 DEGs were obtained as a result of exercise in young groups, including 31 DEGs in SOL YE/YC and 13 DEGs in GAS YE/YC (Table 2). Of the 31 DEGs in SOL YE/YC, 11 genes were upregulated, and 20 were downregulated, whereas among 13 DEGs in GAS YE/YC, seven genes were upregulated, and six were downregulated (Table 2). For each of SOL and GAS, the 30 DEGs with largest absolute value of fold change are plotted (Figure 3A, 3B and Table 2).

**Table 2:** The commonly expressed DEGs for exercise effect in young groups.

**Gene Symbol**	**SOL YE/SOL YC**	***p*.value**	**GAS YE/GAS YC**	***p*.value**
** Up-regulated in both (SOL and GAS)**				
No corresponding gene				
** Down-regulated in both (SOL and GAS)**				
*Nr1d1*	−2.53	0.0254	−1.70	0.0149
				
*Pik3r1* ^*^	1.62	0.0358	−1.54	0.0473

Genes with ^*^ are different pattern between SOL and GAS.

To identify how many of the genes were commonly changed owing to exercise in young groups between SOL and GAS, we checked for the overlapping DEGs in a Venn diagram. The number of DEGs commonly expressed was two (Figure 3C). Of the two common DEGs, nuclear receptor subfamily 1 group D member 1 (*Nr1d1*) exhibited a similar pattern between SOL and GAS, meaning the fold change of YE/YC showed downregulation in both SOL and GAS (downregulation in both SOL YE/YC and GAS YE/YC) (Table 2). In contrast, phosphoinositide-3-kinase regulatory subunit 1 (*Pik3r1*) showed a different pattern between SOL and GAS. Namely, for *Pik3r1*, the fold change of YE/YC exhibited upregulation in SOL, whereas it exhibited downregulation in GAS (upregulation in SOL YE/YC and downregulation in GAS YE/YC) (Table 2).

### Identification of DEGs between OC vs. OE in SOL and GAS and commonly expressed DEGs

**Figure 4:**
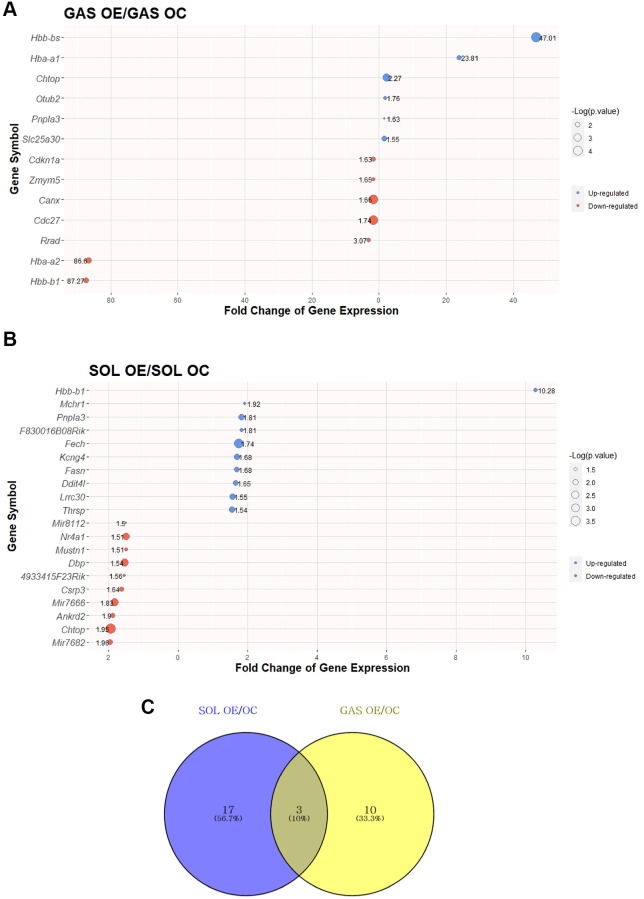
**Top 30 DEGs between “OC vs. OE” in SOL and GAS and Venn diagram to find commonly expressed DEGs.** The point sizes of (**A**) and (**B**) represent ‒Log_10_ (*p*-value). For (**A**) and (**B**), blue points indicate upregulated genes, and red points indicate downregulated genes. (**A**) Top 30 DEGs in GAS OE/OC. (**B**) Top 30 DEGs in SOL OE/OC. (**C**) Venn diagram of DEGs between OC and OE in SOL and GAS. For commonly expressed genes, detailed fold changes and *p*-values are shown in the Table 3.

To elucidate the effect of exercise on SOL and GAS in the old groups, DEG analysis was performed. DEGs were identified following a >1.5-fold change in random expectation (*p* < 0.05). A total of 30 DEGs were obtained by exercise in the old groups, including 20 DEGs in SOL OE/OC and 13 DEGs in GAS OE/OC (Table 3). Out of 20 DEGs in SOL OE/OC, 10 genes were upregulated, and 10 were downregulated, whereas among 13 DEGs in GAS OE/OC, six genes were upregulated, and seven were downregulated (Table 3). For each of SOL and GAS, the 30 DEGs with largest absolute value of fold change are plotted (Figure 4A, 4B).

**Table 3:** The commonly expressed DEGs for exercise effect in old groups.

**Gene Symbol**	**SOL OE/SOL OC**	***p*.value**	**GAS OE/GAS OC**	***p*.value**
** Up-regulated in both (SOL and GAS)**				
*Pnpla3*	1.81	0.0092	1.63	0.0436
** Down-regulated in both (SOL and GAS)**				
No corresponding gene				
*Hbb-b1* ^*^	10.28	0.0280	−87.27	0.0105
*Chtop* ^*^	−1.95	0.0002	2.27	0.0016

Genes with ^*^ are different pattern between SOL and GAS.

To determine how many of the genes were commonly altered by exercise in old groups between SOL and GAS, we explored commonly expressed DEGs between SOL and GAS (DEGs of SOL OE/OC and GAS OE/OC). Three common DEGs were found (Figure 4C), of which patatin-like phospholipase domain-containing 3 (*Pnpla3*) exhibited a similar pattern between SOL and GAS (Table 3). Specifically, for *Pnpla3* the fold change of OE/OC displayed upregulation in SOL and GAS (upregulation in both SOL OE/OC and GAS OE/OC) (Table 3). However, the hemoglobin subunit beta-1 (*Hbb-b1*) and chromatin target of PRMT1 (*Chtop*) showed different patterns (Table 3). For *Hbb-b1*, the fold change of OE/OC exhibited upregulation in SOL, whereas it exhibited downregulation in GAS (upregulation in SOL OE/OC and downregulation in GAS OE/OC) (Table 3). In the case of *Chtop*, the fold change of OE/OC displayed downregulation in SOL but upregulation in GAS (downregulation in SOL OE/OC and upregulation in GAS OE/OC) (Table 3).

### Gene Ontology (GO) term and Kyoto Encyclopedia of Genes and Genomes (KEGG) pathway enrichment analyses using DEGs of SOL/GAS in young and old groups

**Figure 5:**
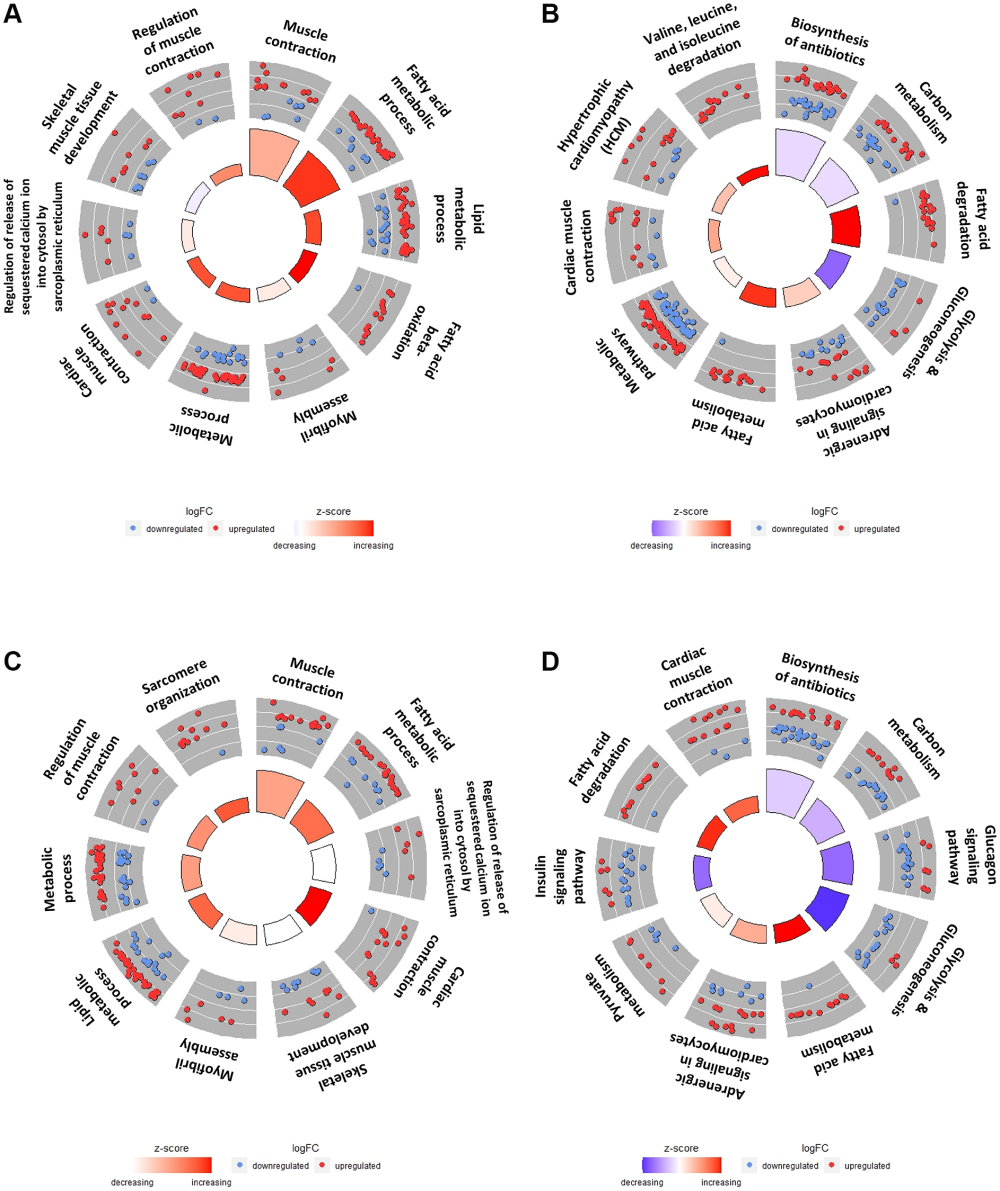
**Top 10 GO terms and KEGG pathways of DEGs in SOL vs. GAS in young and old groups.** The red points indicate upregulated DEGs, while the blue points indicate downregulated DEGs in the outer circle. The z-score is represented by the color of the inner circle. (**A**) Top 10 GO terms using DEGs in the SOL YC/GAS YC. (**B**) Top 10 KEGG pathway plots using DEGs in the SOL YC/GAS YC. (**C**) Top 10 GO terms by DEGs in SOL OC/GAS OC. (**D**) Top 10 KEGG pathway plots of SOL OC/GAS OC. For detailed adjusted *p*-values and z-scores, refer to the Tables 4, 5. For significantly enriched pathway other than top 10, refer to the Supplementary Tables 3–6.

To clarify the differences between SOL and GAS by utilizing DEGs in SOL YC/GAS YC and SOL OC/GAS OC, GO term and KEGG pathway enrichment analyses were performed. The number of DEGs in SOL YC/GAS YC was 1060, while in SOL OC/GAS OC, it was 1052; these DEGs were used for enrichment analyses (Supplementary Table 2). For the top 10 GO terms by SOL YC/GAS YC, the pathways associated with fatty acid metabolism were significantly enriched, including fatty acid beta-oxidation (GO:0006635), fatty acid metabolic process (GO:0006631), and lipid metabolic process (GO:0006629) (Figure 5A and Table 4). For the top 10 KEGG pathways in the young groups, the enrichment scores of fatty acid degradation (mmu00071) and fatty acid metabolism (mmu01212) were significant (Figure 5B and Table 5). Regarding valine, leucine, and isoleucine degradation (mmu00280), this pathway had only 14 upregulated DEGs in SOL YC/GAS YC (Table 5). In addition, out of 19 DEGs of glycolysis/gluconeogenesis (mmu00010), 15 genes were downregulated, and four were upregulated (Table 5).

**Table 4:** The significant enriched GO terms of DEGs between SOL YC vs. GAS YC.

**ID**	**Term**	***z*-score**	**Adjusted *p*.value**
GO:0006936	Muscle contraction	1.34	0
GO:0006631	Fatty acid metabolic process	2.96	0
GO:0006629	Lipid metabolic process	2.77	0.0001
GO:0006635	Fatty acid beta-oxidation	3.21	0.0001
GO:0030239	Myofibril assembly	0.33	0.0001
GO:0008152	Metabolic process	2.66	0.0001
GO:0060048	Cardiac muscle contraction	2.67	0.0002
GO:0010880	Regulation of release of sequestered calcium ion into cytosol by sarcoplasmic reticulum	0.33	0.0002
GO:0007519	Skeletal muscle tissue development	−0.26	0.0002
GO:0006937	Regulation of muscle contraction	1.90	0.0002

**Table 5:** The significant enriched KEGG pathway of DEGs between SOL YC vs. GAS YC.

**ID**	**Term**	***z*-score**	**Adjusted *p*.value**
mmu01130	Biosynthesis of antibiotics	−0.60	0
mmu01200	Carbon metabolism	−0.56	0
mmu00071	Fatty acid degradation	3.77	0
mmu00010	Glycolysis/Gluconeogenesis	−2.52	0
mmu04261	Adrenergic signaling in cardiomyocytes	0.96	0
mmu01212	Fatty acid metabolism	3.5	0
mmu01100	Metabolic pathways	0.29	0
mmu04260	Cardiac muscle contraction	1.70	0.0001
mmu05410	Hypertrophic cardiomyopathy (HCM)	1.21	0.0001
mmu00280	Valine, leucine and isoleucine degradation	3.74	0.0001

For the top 10 GO terms by SOL OC/GAS OC, the gene set related to lipid metabolism, such as fatty acid metabolic process (GO:0006631) and lipid metabolic process (GO:0006629), was significantly enriched (Figure 5C and Table 6). Regarding the top 10 KEGG pathways by SOL OC/GAS OC, the enrichment scores of fatty acid metabolism (mmu01212) and fatty acid degradation (mmu00071) were significant (Figure 5D and Table 7). For pathways associated with glucose metabolism, three gene sets belonged to the top 10 KEGG pathways in the old groups (Figure 5D and Table 7). These pathways included glucagon signaling (mmu04922), glycolysis/gluconeogenesis (mmu00010), and insulin signaling (mmu04910) (Figure 5D and Table 7).

**Table 6:** The significant enriched GO terms of DEGs between SOL OC vs. GAS OC.

**ID**	**Term**	***z*-score**	**Adjusted *p*.value**
GO:0006936	Muscle contraction	1.61	0
GO:0006631	Fatty acid metabolic process	2.41	0
GO:0010880	Regulation of release of sequestered calcium ion into cytosol by sarcoplasmic reticulum	0	0
GO:0060048	Cardiac muscle contraction	3.36	0
GO:0007519	Skeletal muscle tissue development	0	0.0001
GO:0030239	Myofibril assembly	0.33	0.0001
GO:0006629	Lipid metabolic process	2.55	0.0001
GO:0008152	Metabolic process	1.70	0.0001
GO:0006937	Regulation of muscle contraction	1.90	0.0002
GO:0045214	Sarcomere organization	2.71	0.0004

**Table 7:** The significant enriched KEGG pathway of DEGs between SOL OC vs. GAS OC.

**ID**	**Term**	***z*-score**	**Adjusted *p*.value**
mmu01130	Biosynthesis of antibiotics	−0.67	0
mmu01200	Carbon metabolism	−1.04	0
mmu04922	Glucagon signaling pathway	−1.96	0
mmu00010	Glycolysis/Gluconeogenesis	−2.67	0
mmu01212	Fatty acid metabolism	3.05	0.0003
mmu04261	Adrenergic signaling in cardiomyocytes	1.28	0.0003
mmu00620	Pyruvate metabolism	0.30	0.0005
mmu04910	Insulin signaling pathway	−1.96	0.0006
mmu00071	Fatty acid degradation	2.89	0.0006
mmu04260	Cardiac muscle contraction	2.32	0.0006

### Gene set enrichment analysis (GSEA) for apoptosis in SOL and GAS

**Figure 6:**
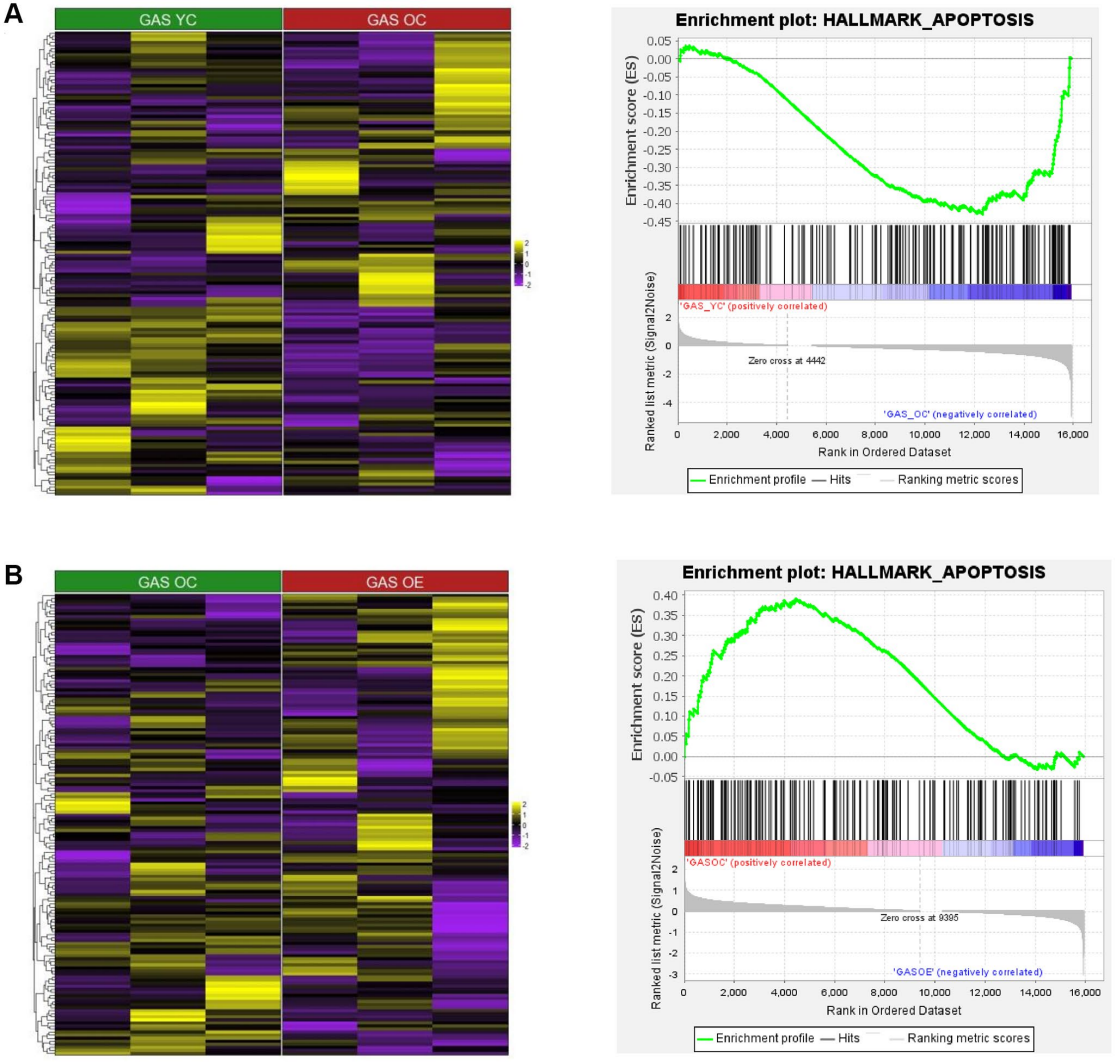
**Heatmap and GSEA results for HALLMARK_APOPTOSIS in “YC vs. OC”, “YC vs. YE”, and “OC vs. OE” in SOL and GAS.** The heatmap and enrichment plot in (**A**) GAS YC vs. GAS OC and (**B**) GAS OC vs. GAS OE. For the color of the heatmap, yellow indicates upregulated genes, and purple indicates downregulated genes. The significant GSEA results for gene sets other than HALLMARK_APOPTOSIS are listed in Tables 8–13.

To investigate the association between aging and exercise and apoptosis in SOL and GAS, we analyzed data at the gene set levels. For the comparison between young and old groups, HALLMARK_APOPTOSIS pathway was more enriched in GAS OC than in GAS YC (Figure 6A and Table 8), whereas in SOL, the enrichment score (ES) between SOL YC and SOL OC was not significant (ES = 0.36, *p* = 0.099). For the comparison between sedentary control and exercise in old groups, the HALLMARK_APOPTOSIS pathway was more enriched in GAS OC than in GAS OE (Figure 6B and Table 9). In SOL, the enrichment score between SOL OC and SOL OE was not significant (ES = −0.25, *p* = 0.615). In the young groups, there were no significant difference between the control and exercise groups in both SOL and GAS.

**Table 8:** The results of GSEA (GAS YC vs. GAS OC).

**Group**	**Name of gene set**	**Size**	**ES**	**NES**	***p*.value**	**FDR**
**Enriched in GAS YC**					
1	HALLMARK_EPITHELIAL_MESENCHYMAL_TRANSITION	195	0.33	1.74	0	0.0130
2	HALLMARK_ANGIOGENESIS	36	0.42	1.55	0	0.0159
3	HALLMARK_UV_RESPONSE_DN	143	0.27	1.27	0	0.1419
1	HALLMARK_FATTY_ACID_METABOLISM	149	−0.45	−1.38	0.0060	0.1990
**Enriched in GAS OC**					
2	HALLMARK_IL6_JAK_STAT3_SIGNALING	83	−0.46	−1.35	0.0260	0.1603
3	HALLMARK_KRAS_SIGNALING_UP	195	−0.43	−1.33	0.0010	0.1678
4	HALLMARK_APOPTOSIS	161	−0.43	−1.32	0.0181	0.1821
5	HALLMARK_OXIDATIVE_PHOSPHORYLATION	198	−0.42	−1.31	0.0070	0.1711
6	HALLMARK_INFLAMMATORY_RESPONSE	189	−0.42	−1.30	0.0190	0.1700

Size, the number of corresponding genes to gene sets. Abbreviations: ES: Enrichment Score; NES: Normalized Enrichment Score.

**Table 9:** The results of GSEA (GAS OC vs. GAS OE).

**Group**	**Name of gene set**	**Size**	**ES**	**NES**	***p*.value**	**FDR**
**Enriched in GAS OC**					
1	HALLMARK_UV_RESPONSE_DN	143	0.47	1.66	0	0.0301
2	HALLMARK_TNFA_SIGNALING_VIA_NFKB	195	0.46	1.64	0	0.0202
3	HALLMARK_PROTEIN_SECRETION	95	0.48	1.63	0	0.0148
4	HALLMARK_UNFOLDED_PROTEIN_RESPONSE	112	0.46	1.58	0	0.0214
5	HALLMARK_MTORC1_SIGNALING	199	0.42	1.53	0.0021	0.0339
6	HALLMARK_ANDROGEN_RESPONSE	97	0.45	1.51	0.0045	0.0357
7	HALLMARK_G2M_CHECKPOINT	196	0.41	1.49	0.0000	0.0383
8	HALLMARK_E2F_TARGETS	200	0.41	1.49	0.0000	0.0343
9	HALLMARK_INFLAMMATORY_RESPONSE	189	0.41	1.46	0.0021	0.0464
10	HALLMARK_UV_RESPONSE_UP	155	0.40	1.41	0.0098	0.0746
11	HALLMARK_TGF_BETA_SIGNALING	54	0.45	1.40	0.0368	0.0758
12	HALLMARK_MITOTIC_SPINDLE	198	0.38	1.39	0.0073	0.0742
13	HALLMARK_APOPTOSIS	161	0.39	1.39	0.0151	0.0720
14	HALLMARK_MYC_TARGETS_V1	200	0.38	1.37	0.0104	0.0786
**Enriched in GAS OE**					
1	HALLMARK_INTERFERON_ALPHA_RESPONSE	95	−0.46	−1.96	0	0.0004
2	HALLMARK_INTERFERON_GAMMA_RESPONSE	192	−0.33	−1.61	0	0.0051
3	HALLMARK_MYOGENESIS	198	−0.27	−1.26	0.0303	0.0628

Size, the number of corresponding genes to gene sets. Abbreviations: ES: Enrichment Score; NES: Normalized Enrichment Score.

### GSEA analysis for myogenesis in SOL and GAS

**Figure 7:**
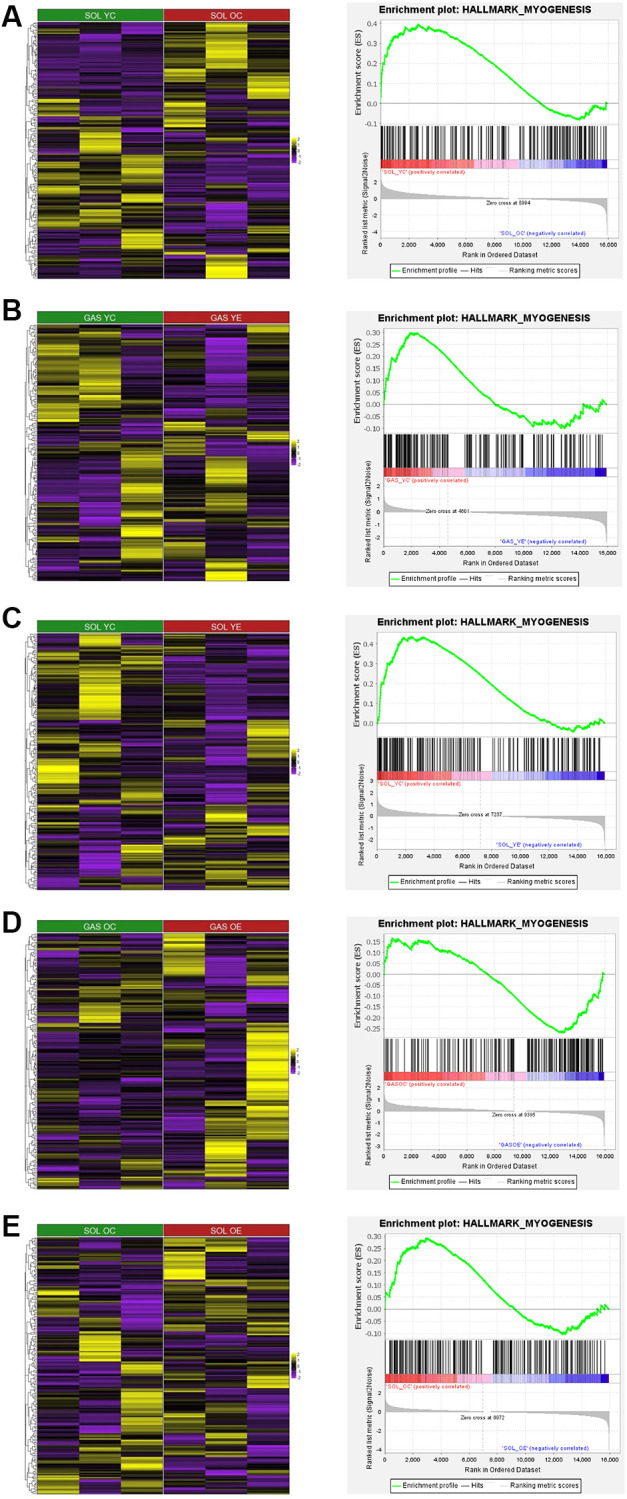
**Heatmap and GSEA results for HALLMARK_MYOGENESIS in “YC vs. OC”, “YC vs. YE”, and “OC vs. OE” in SOL and GAS.** The heatmap and enrichment plot between (**A**) SOL YC vs. SOL OC, (**B**) GAS YC vs. GAS YE, (**C**) SOL YC vs. SOL YE, (**D**) GAS OC vs. GAS OE, and (**E**) SOL OC vs. SOL OE. For the color of the heatmap, yellow indicates upregulated genes, and purple indicates downregulated genes. The significant results of GSEA other than HALLMARK_MYOGENESIS are listed in Tables 8–13.

To elucidate the relationship between aging and exercise and myogenesis in SOL and GAS, we conducted GSEA. For the comparison between young and old groups, HALLMARK_MYOGENESIS pathway was more enriched in SOL YC than in SOL OC (Figure 7A and Table 10), whereas in GAS, the enrichment score between GAS YC and GAS OC was not significant (ES = −0.25, *p* = 0.955). For the results of GSEA using sedentary control and exercise in young groups, the HALLMARK_MYOGENESIS pathway was more enriched in GAS YC than in GAS YE (Figure 7B and Table 11). Similar to GAS, this pathway was also more enriched in SOL YC than in SOL YE (Figure 7C and Table 12). For the comparison between control and exercise in old groups, HALLMARK_MYOGENESIS pathway was more enriched in GAS OE than in GAS OC (Figure 7D and Table 9), whereas in SOL, it was more enriched in OC groups than in OE groups (Figure 7E and Table 12).

**Table 10:** The results of GSEA (SOL YC vs. SOL OC).

**Group**	**Name**	**Size**	**ES**	**NES**	***p*.value**	**FDR**
**Enriched in SOL YC**					
1	HALLMARK_EPITHELIAL_MESENCHYMAL_TRANSITION	195	0.70	2.38	0	0
2	HALLMARK_ALLOGRAFT_REJECTION	186	0.59	1.98	0	0
3	HALLMARK_ANGIOGENESIS	36	0.67	1.86	0	0
4	HALLMARK_INTERFERON_GAMMA_RESPONSE	192	0.52	1.74	0	0.0008
5	HALLMARK_IL2_STAT5_SIGNALING	194	0.50	1.69	0	0.0027
6	HALLMARK_APICAL_JUNCTION	190	0.50	1.68	0	0.0026
7	HALLMARK_INTERFERON_ALPHA_RESPONSE	95	0.52	1.65	0	0.0034
8	HALLMARK_INFLAMMATORY_RESPONSE	189	0.48	1.61	0	0.0046
9	HALLMARK_IL6_JAK_STAT3_SIGNALING	83	0.51	1.61	0	0.0044
10	HALLMARK_KRAS_SIGNALING_UP	195	0.46	1.57	0.0010	0.0075
11	HALLMARK_COMPLEMENT	186	0.45	1.53	0.0010	0.0123
12	HALLMARK_COAGULATION	120	0.47	1.52	0.0044	0.0140
13	HALLMARK_APICAL_SURFACE	42	0.53	1.49	0.0184	0.0180
14	HALLMARK_HYPOXIA	193	0.40	1.36	0.0093	0.0869
15	HALLMARK_MTORC1_SIGNALING	199	0.40	1.36	0.0125	0.0891
16	HALLMARK_MYOGENESIS	198	0.40	1.33	0.0166	0.1123
17	HALLMARK_ESTROGEN_RESPONSE_EARLY	188	0.38	1.28	0.0491	0.1631
**Enriched in SOL OC**					
There was no geneset that satisfied the following criteria (*p* value < 0.05, FDR < 0.25)						

Size, the number of corresponding genes to gene sets. Abbreviations: ES: Enrichment Score; NES: Normalized Enrichment Score.

**Table 11:** The results of GSEA (GAS YC vs. GAS YE).

**Group**	**Name**	**Size**	**ES**	**NES**	***p*.value**	**FDR**
**Enriched in GAS YC**					
1	HALLMARK_G2M_CHECKPOINT	196	0.26	1.40	0	0.1262
2	HALLMARK_MYOGENESIS	198	0.30	1.36	0	0.0856
**Enriched in GAS YE**					
1	HALLMARK_OXIDATIVE_PHOSPHORYLATION	198	−0.60	−1.92	0	0
2	HALLMARK_INTERFERON_ALPHA_RESPONSE	95	−0.61	−1.86	0	0
3	HALLMARK_INTERFERON_GAMMA_RESPONSE	192	−0.55	−1.77	0	0
4	HALLMARK_FATTY_ACID_METABOLISM	149	−0.51	−1.63	0	0.0032
5	HALLMARK_BILE_ACID_METABOLISM	103	−0.50	−1.56	0	0.0097
6	HALLMARK_ADIPOGENESIS	199	−0.46	−1.49	0	0.0216
7	HALLMARK_CHOLESTEROL_HOMEOSTASIS	73	−0.45	−1.38	0.0346	0.0864
8	HALLMARK_XENOBIOTIC_METABOLISM	180	−0.42	−1.34	0.0101	0.1237

Size, the number of corresponding genes to gene sets. Abbreviations: ES: Enrichment Score; NES: Normalized Enrichment Score.

**Table 12:** The results of GSEA (SOL YC vs. SOL YE).

**Group**	**Name**	**Size**	**ES**	**NES**	***p*.value**	**FDR**
**Enriched in SOL YC**					
1	HALLMARK_EPITHELIAL_MESENCHYMAL_TRANSITION	195	0.63	2.45	0	0
2	HALLMARK_ANGIOGENESIS	36	0.65	1.92	0	0.0010
3	HALLMARK_MYOGENESIS	198	0.44	1.71	0	0.0050
4	HALLMARK_ALLOGRAFT_REJECTION	186	0.42	1.66	0	0.0079
5	HALLMARK_APICAL_JUNCTION	190	0.42	1.65	0	0.0067
6	HALLMARK_TNFA_SIGNALING_VIA_NFKB	195	0.42	1.64	0	0.0070
7	HALLMARK_INFLAMMATORY_RESPONSE	189	0.42	1.64	0	0.0065
8	HALLMARK_IL6_JAK_STAT3_SIGNALING	83	0.46	1.63	0.0063	0.0059
9	HALLMARK_TGF_BETA_SIGNALING	54	0.50	1.62	0.0034	0.0063
10	HALLMARK_UV_RESPONSE_DN	143	0.40	1.52	0.0015	0.0205
11	HALLMARK_HEDGEHOG_SIGNALING	36	0.50	1.52	0.0259	0.0191
12	HALLMARK_KRAS_SIGNALING_UP	195	0.38	1.50	0.0015	0.0212
13	HALLMARK_APICAL_SURFACE	42	0.47	1.45	0.0307	0.0333
14	HALLMARK_IL2_STAT5_SIGNALING	194	0.35	1.35	0.0159	0.0836
15	HALLMARK_COMPLEMENT	186	0.34	1.32	0.0246	0.1066
16	HALLMARK_HYPOXIA	193	0.33	1.30	0.0428	0.1215
**Enriched in SOL YE**					
1	HALLMARK_OXIDATIVE_PHOSPHORYLATION	198	−0.60	−2.62	0	0
2	HALLMARK_INTERFERON_ALPHA_RESPONSE	95	−0.57	−2.24	0	0
3	HALLMARK_ADIPOGENESIS	199	−0.48	−2.03	0	0
4	HALLMARK_INTERFERON_GAMMA_RESPONSE	192	−0.40	−1.71	0	0.0046
5	HALLMARK_FATTY_ACID_METABOLISM	149	−0.40	−1.63	0	0.0069
6	HALLMARK_MYC_TARGETS_V1	200	−0.34	−1.45	0	0.0383
7	HALLMARK_XENOBIOTIC_METABOLISM	180	−0.34	−1.45	0.0033	0.0329
8	HALLMARK_BILE_ACID_METABOLISM	103	−0.35	−1.37	0.0224	0.0588

Size, the number of corresponding genes to gene sets. Abbreviations: ES: Enrichment Score; NES: Normalized Enrichment Score.

**Table 13:** The results of GSEA (SOL OC vs. SOL OE).

**Group**	**Name**	**SIZE**	**ES**	**NES**	***p*.value**	**FDR**
**Enriched in SOL OC**					
1	HALLMARK_OXIDATIVE_PHOSPHORYLATION	198	0.42	1.81	0	0.0077
2	HALLMARK_TNFA_SIGNALING_VIA_NFKB	195	0.40	1.74	0	0.0093
3	HALLMARK_ANGIOGENESIS	36	0.54	1.73	0.0066	0.0074
4	HALLMARK_TGF_BETA_SIGNALING	54	0.48	1.70	0	0.0092
5	HALLMARK_UNFOLDED_PROTEIN_RESPONSE	112	0.37	1.48	0.0119	0.0389
6	HALLMARK_HYPOXIA	193	0.32	1.37	0.0103	0.0913
7	HALLMARK_MYOGENESIS	198	0.29	1.26	0.0191	0.1807
**Enriched in SOL OE**					
1	HALLMARK_ALLOGRAFT_REJECTION	186	−0.50	−1.88	0	0.0008
2	HALLMARK_INTERFERON_GAMMA_RESPONSE	192	−0.46	−1.73	0	0.0039
3	HALLMARK_INTERFERON_ALPHA_RESPONSE	95	−0.50	−1.71	0	0.0036
4	HALLMARK_COMPLEMENT	186	−0.36	−1.37	0.0161	0.2319
5	HALLMARK_KRAS_SIGNALING_UP	195	−0.36	−1.36	0.0213	0.1926

Size, the number of corresponding genes to gene sets. Abbreviations: ES: Enrichment Score; NES: Normalized Enrichment Score.

## DISCUSSION

Muscle fiber type interacts with aging and exercise; aging has a major impact on type II fibers in terms of atrophy, and exercise induces hypertrophy to a different extent depending on fiber type [2, 9]. As such, many researchers have scrutinized the interaction of muscle fiber type with aging and exercise. However, large sets of RNA-sequencing data have rarely been analyzed to clarify the relationship between these factors. Therefore, by utilizing such data and bioinformatics, we attempted in the current study to elucidate how the muscle fiber type interacts with aging and exercise at the genetic level.

### Effect of muscle fiber type on gene expression is greater than that of aging and exercise

PCA plots and heatmaps indicate that the separation by muscle fiber type is most conspicuous among the clusters by muscle fiber type, aging, and exercise. For separation by fiber type, SOL was divided from GAS by PC1 (69.2% of total variance) (Figure 1B). Prior studies performing PCA confirmed that the gene expression of fast-twitch muscles is different from that of slow-twitch muscles by PC1 (46% of total variance) [27], which our findings are compatible with. However, although both EDL and psoas are mainly composed of fast-twitch fibers, EDL exhibited clear separation from psoas by PC2, accounting for 27% of the total variance [27]. This result suggests that in addition to muscle fiber type distribution, other factors may have a significant effect on gene expression. Since the whole muscle lysates used in this study may include blood vessels, connective tissues, and nerves in addition to muscle fibers, EDL may display different gene expression patterns from psoas owing to differences in these factors. Therefore, to elucidate the effects of muscle fiber type on gene expression more clearly, microgenomic profiles, such as single-cell sample analysis, may have to be performed [28]. After the muscle fiber type, clusters by aging were the most prominent. The young and old groups were noticeably separated by PC2 (5.5%) (Figure 1C). In addition, previous data have shown that metabolomics datasets are remarkably clustered into young and old groups [29], which our results agree with.

In the current study, the exercise groups were not clearly divided (Figure 1B, 1C). According to previous research performing PCA, microarray data were prominently separated into exercise and sedentary groups [30]. Presumably, these conflicting results between our study and the one performed previously may stem from the differences in the period of exercise intervention and tissues used in gene expression analysis. For the period of exercise, our protocol required 4 weeks, while exercise intervention in the prior study lasted for 7 weeks. To determine whether exercise for 4 weeks is long enough to elicit the differences in gene expression profiles, further research should be conducted. Furthermore, the experiment in our study relied on skeletal muscles such as SOL and GAS, whereas the left ventricle was utilized in the previous research [30]. To identify whether the cardiac muscle is more responsive than SOL and GAS when intervening with the same exercise regimen, a follow-up study is needed.

### Common DEGs with aging are related to apoptosis, circadian rhythm, and immune signaling

We performed DEG analysis in SOL and GAS to elucidate the differences due to aging. There were 29 common DEGs that were associated with aging (Figure 2C), of which cell death-inducing DNA fragmentation factor alpha like effector A (*Cidea*) was upregulated in both SOL and GAS with age (Table 1). *Cidea* has been confirmed to be involved in apoptosis and lipid metabolism [31–33]. In the skeletal muscle of rats, apoptotic signaling was enhanced, and *Cidea* expression was significantly increased after burn injury [31]. For lipid metabolism, *Cidea*-knockout mice have a lean phenotype, whereas mice with induced transgenic expression of *Cidea* exhibit a healthy obese phenotype [32, 33]. Although it has been confirmed that *Cidea* expression is decreased by aging in adipose tissue [34], it is not known whether *Cidea* expression changes with aging in skeletal muscle. To clarify what influence the alteration of *Cidea* expression in skeletal muscles has on phenotype, further studies are necessary. In addition, collagen genes (*Col1a1, Col1a2, Col3a1, Col4a2, Col5a1, Col5a2, Col5a3*, and *Col6a1*) were downregulated in both SOL and GAS with aging. Prior studies also observed that the concentration of collagen was decreased by aging [35, 36].

D-box-binding PAR BZIP transcription factor (*Dbp*) and *Nr1d1* (also known as Rev-erb-alpha) were downregulated in both SOL and GAS and are referred to as “clock-controlled genes” (Table 1). Considering that reduced *Dbp* expression is correlated with the early aging phenotype in mice [37], there seems to be an interaction between *Dbp* and aging. Furthermore, overexpression of *Nr1d1* increases mitochondrial function and exercise capacity [38]. In contrast, the deficiency of *Nr1d1* leads to the upregulation of atrophy-related genes and increases skeletal muscle atrophy [39]. As we have confirmed here that *Nr1d1* expression is decreased in skeletal muscles owing to aging (Table 1), further investigation is needed to scrutinize the interaction between *Nr1d1* and muscle atrophy induced by aging. However, circadian genes exhibit differences in expression depending on the timing of measurement, tissues, and species [40]; therefore, it is necessary to consider these factors as well.

*Cd74* and *Tyrobp* (also known as *Dap12*) display different expression patterns between SOL and GAS by the effect of aging (Table 1). Importantly, these genes are involved in immune functions. Migration inhibitory factor (the primary ligand of *Cd74*) knockout mice reported to have lived markedly longer than control mice [41]. *Tyrobp* is mainly expressed on innate immune cells, including monocytes, neutrophils, dendritic cells (DCs), and natural killer (NK) cells [42]. Because *Cd74* and *Tyrobp* were barely addressed in terms of aging and exercise effects, it is meaningful to determine whether the differences in their expression between SOL and GAS elicit dissimilar characteristics of immune function.

### Common DEGs with exercise are associated with circadian rhythm and insulin signaling in young groups and hemoglobin subunit in old groups

In young groups, there were two DEGs commonly expressed owing to exercise: *Nr1d1* and *Pi3kr1* (Table 2). In our study, *Nr1d1* expression was downregulated in both SOL and GAS (Table 2). However, as prior research has shown that acute exercise increases *Nr1d1* expression [43], our findings seem to contradict these previous data. Putatively, these inconsistent results may be due to differences in the exercise period (acute vs. chronic). Considering that *Nr1d1* has a positive correlation with mitochondrial function and oxidative capacity in skeletal muscles, the increase in *Nr1d1* expression by acute exercise may be due to the need to temporarily improve mitochondrial function. On the other hand, the reason chronic exercise increases *Nr1d1* expression may be because chronic exercise increases oxidative capacity so the exercise group can possibly maintain homeostasis without the influence of *Nr1d1*. Nonetheless, the correlation between *Nr1d1* and exercise period remains to be explored. *Pi3kr1* displayed different expression patterns in SOL and GAS as a result of exercise (Table 2). For instance, *Pi3kr1* expression was upregulated in SOL and downregulated in GAS (Table 2). According to a previous study, *Pi3kr1* is indispensable for insulin signaling, and the selective deletion of *Pi3kr1* augmented insulin sensitivity in fast-twitch muscle (EDL) but not in SOL [44]. As the effect of exercise on glucose uptake differs depending on muscle fiber type composition [45], further research is warranted to determine whether the differences in *Pi3kr1* expression partially contribute to the differences in insulin resistance according to muscle fiber type.

In old groups with exercise, common DEGs included *Pnpla3* and *Hbb-b1* (Table 3). *Pnpla3* expression was upregulated in both SOL and GAS (Table 3). However, as *Pnpla3* is mainly located in the liver and retina [46], the alteration of *Pnpla3* expression in skeletal muscle does not seem to be important. *Hbb-b1*, on the other hand, showed different patterns between SOL and GAS (Table 3). With exercise, *Hbb-b1* expression was upregulated in SOL and downregulated in GAS (Table 3). *Hbb-b1* is linked to the formation of hemoglobin, and the mutation of *Hbb-b1* is associated with abnormal oxygen transportation [47]. As *Hbb-b1* is involved in oxygen transportation and exhibits the most prominent differences between SOL and GAS, further studies should be conducted to clarify the interaction between this protein and muscle fiber type.

### Differences in metabolism according to muscle fiber type and significant upregulation of BCAA degradation in SOL compared to that in GAS

To identify the differences between SOL and GAS, we performed GO analysis using DEGs between these two muscle types. For GO and KEGG pathways in all age groups, fatty acid metabolism was upregulated in SOL, while gene sets associated with glucose metabolism were downregulated (Figure 5A–5D and Tables 4–7). Considering that the enzyme activities of fatty acid metabolism are higher in slow-twitch muscle than in fast-twitch muscle and that glucose metabolism is invigorated in fast-twitch muscle [48], our findings are compatible with those shown previously. For the KEGG pathway in young groups, the valine, leucine, and isoleucine degradation pathways were significantly upregulated in SOL (Figure 5B and Table 5). Considering that branched-chain amino acid (BCAA) catabolism leads to a decrease in myofiber size [49], there is a need to study whether the difference in BCAA degradation elicits different patterns of atrophy based on muscle fiber type.

### Necessity for different interventions depending on the muscle fiber type due to different patterns of apoptosis and myogenesis with aging

In fast-twitch muscle, our results showed that the apoptotic pathway was significantly enriched in the old group compared to the young group (Figure 6A and Table 8), whereas no significant difference was observed with aging in terms of myogenesis. Considering that the muscle mass-to-body weight ratio is decreased in fast-twitch muscle with age [50], the cause of atrophy may be largely owing to changes in the apoptotic process due to aging, not myogenesis. Unlike GAS, no significant difference in the apoptotic process due to aging was seen in SOL, while myogenesis was significantly enriched in the young group compared to the old group (Figure 7A). According to a prior study, the cross-sectional area of slow-twitch muscles, such as SOL, is decreased by aging in rats [51]. Given the results of our study, atrophy of slow-twitch muscle may primarily result from the alteration of myogenesis with age and not from the change in apoptosis. As the cause of atrophy by aging seems to differ depending on muscle fiber type composition, appropriate intervention according to fiber type should be considered. As the interaction between aging-induced atrophy and muscle fiber type has rarely been investigated regarding both apoptosis and myogenesis, further research is needed to clarify the differences in these two processes by aging depending on fiber type at the cellular level.

### Mitigation of apoptosis and augmentation of myogenesis in old groups by chronic aerobic exercise in GAS but not in SOL

We found that exercise intervention for 4 weeks had different effects on the apoptosis and myogenesis depending on the muscle fiber type. In old groups, the apoptotic pathway was significantly enriched in the sedentary control compared to the exercise group in GAS (Figure 6B and Table 9), whereas no significant difference was seen in SOL. Our results are consistent with the results of previous research, which show that exercise significantly attenuates apoptosis in fast-twitch muscle but not in slow-twitch [52]. According to prior study, age-related muscle atrophy mainly relies on intrinsic apoptotic pathway and this process are different between slow and fast-twitch muscle [51, 53–56]. In detail, caspase-9, which is a key molecule in the intrinsic apoptotic pathway, were significantly up-regulated in fast-twitch muscle by aging, whereas were not in slow-twitch muscle [50]. Since it has been not determined whether chronic exercise results in down-regulation of apoptosis by modulating the amount and activity of caspase-9 in fast-twitch muscle, further study need to be performed.

In the young groups, there was no significant difference due to exercise in either SOL or GAS. Previous research has shown that chronic exercise induces different apoptotic processes depending on the muscle fiber type in 4-week-old rats [57]. Putatively, the difference in our results with those found previously may be due to the differences in the type of exercise (concentric vs. eccentric). The exercise protocol in the prior study relied on eccentric exercise by decreasing the slope of the treadmill [57], while our protocol calls for a 6° incline. As eccentric exercise elicits more muscle damage than concentric exercise [58], previous studies using eccentric exercise may have exhibited more conspicuous differences in the apoptotic process depending on muscle fiber type than our current study. Whether these conflicting results stem from the differences in the type of exercise remains to be investigated.

In the old groups, myogenesis was more enriched in exercise groups than in the sedentary control in GAS (Figure 7D and Table 9), whereas there were no significant differences seen in SOL. Our results are consistent with those shown previously where 8 weeks of treadmill exercise significantly increase the level of the myogenin, transcription factor involved in myogenesis, in fast-twitch muscle but not in slow-twitch muscle in aged rats [59]. Considering that myogenesis is involved in muscle regeneration [60], a follow-up study should be performed to identify whether chronic treadmill exercise enhances the regenerative capacity of fast-twitch muscles more so than slow-twitch muscles in old groups.

In the young groups, myogenesis was not more enriched in the exercise group than in the sedentary group in both SOL and GAS (Figure 7B, 7C, Tables 11 and 13). According to a previous study, treadmill exercise for 13 weeks doesn’t induce to significant increase in myogenic clones of GAS in young group, whereas myogenic clones was significantly elevated in old group [61]. Therefore, aerobic exercise can mitigate the decrease in myogenesis by aging, but does not seem to up-regulate myogenic pathway more actively in young group. Since there are not many studies done in this regard, further studies are needed to clarify. Taken together, future related studies should be mindful that the interaction of muscle fiber type with aging and exercise is relevant with respect to apoptosis and myogenesis.

## CONCLUSION

There is a myriad of conflicting results from studies on exercise and aging. As research scientists, we are obligated to identify the causes of inconsistent outcomes in scientific experiments. In this study, we demonstrated that gene expression patterns differ significantly according to muscle fiber type (slow-twitch vs. fast-twitch), making it imperative that this be considered when investigating exercise and aging. Furthermore, to contribute to taking a step closer to a unanimous conclusion, elucidating how exercise frequency, intensity, time, type, and intervention period interact with muscle fiber type distribution would be invaluable.

This study has limitation. SOL and GAS exhibits different activation patterns with increasing demands of force and speed [62]. In detail, as exercise intensity increased, the EMG amplitude ratios of SOL to GAS decreased [62]. Thus, in order to exclude mechanical differences, exercise is performed at an intensity that induced the same level of activation of SOL and GAS.

The data discussed in this publication have been deposited in NCBI’s Gene Expression Omnibus and are accessible through GEO Series accession number GSE198266 (https://www.ncbi.nlm.nih.gov/geo/query/acc.cgi?acc=GSE198266).

## MATERIALS AND METHODS

### Animals

All procedures were based on previously published data [63]. Briefly, the 9-week-old C57BL/6 young male mice and 84-week-old mice were purchased from Central Lab. The groups were divided into young control (YC), young exercise (YE), old control (OC), and old exercise (OE) groups. Mice were individually housed in standard conditions with food and water ad libitum. Each group comprised three mice and all mice were subjected to RNA sequencing analysis. Details on animal care were provided in a previous study [63].

### Exercise protocol

Before 4 weeks of treadmill exercise, adaptation exercise was conducted once a day for 3 days. Mice were familiarized with the treadmill for 15 min/session at a 0 m/min for 3 min, 5 m/min for 2 min and 8 m/min for 10 min and 6° incline. In 4 weeks of treadmill exercise, mice allocated to perform treadmill running were subject to 6° incline and for warm up, 2 min speed of 0 m/min, speed of 5 m/min, 8 m/min, 10 m/min for 1 min each, and then 12 m/min for 30 min at first week, 2 m/min were increased every week, and cool down at 5 m/min for 2 min for 1 session (37 min). 2 session/day were performed. Between the session, there were at least 1 h break time was given.

### SOL and GAS RNA extraction

All procedures were based on a previous study [63]. Briefly, total RNA was extracted from SOL and GAS of mice using a total RNA extraction kit (Ribozol, Amresco) following the manufacturer’s protocol. The remaining processes, including cDNA synthesis, check for purity and integrity were presented in previously published data [63].

### RNA-Seq library preparation and sequencing

Total RNA concentration was calculated by Quant-IT RiboGreen (Invitrogen, #R11490). To assess the integrity of the total RNA, samples are run on the TapeStation RNA screentape (Agilent, #5067–5576). Only high-quality RNA preparations, with RIN greater than 7.0, were used for RNA library construction. A library was independently prepared with 1ug of total RNA for each sample by Illumina TruSeq Stranded mRNA Sample Prep Kit (Illumina, Inc., San Diego, CA, USA, #RS-122-2101). The first step in the workflow involves purifying the poly-A containing mRNA molecules using poly-T-attached magnetic beads. Following purification, the mRNA is fragmented into small pieces using divalent cations under elevated temperature. The cleaved RNA fragments are copied into first strand cDNA using SuperScript II reverse transcriptase (Invitrogen, #18064014) and random primers. This is followed by second strand cDNA synthesis using DNA Polymerase I, RNase H and dUTP. These cDNA fragments then go through an end repair process, the addition of a single ‘A’ base, and then ligation of the adapters. The products are then purified and enriched with PCR to create the final cDNA library. The libraries were quantified using KAPA Library Quantification kits for Illumina sequencing platforms according to the qPCR Quantification Protocol Guide (KAPA BIOSYSTEMS, #KK4854) and qualified using the TapeStation D1000 ScreenTape (Agilent Technologies, #5067–5582). Indexed libraries were then submitted to an Illumina NovaSeq (Illumina, Inc., San Diego, CA, USA), and the paired-end (2 × 100 bp) sequencing was performed by the Macrogen Incorporated.

### Data pre-processing and quality check

Raw data (FPKM) were processed using the Bioconductor preprocessCore package with default parameters for filtering, signal +1 and logarithm transformation, and quantile normalization [64]. DEG analysis was performed using fold change calculation and the Bioconductor genefilter package [65].

### Bioinformatics tools

#### PCA analysis, heatmap, and Venn diagram

PCA was performed using the built-in R function prcomp() [66]. The Github ggbiplot and ggplot2 packages were used to plot a PCA graph [67]. Pre-processed data were used for PCA. The Bioconductor ComplexHeatmap package was utilized to create a heatmap [68]. For the heatmap in Figure 1, pre-processed data were screened based on the following criteria: After the median value and standard deviation (SD) for each gene were calculated using the *log*_2_ expression value, genes with a median value of <5 and SD of the bottom 25% were excluded from the analysis. Of the 18,795 total genes, 1268 passed this screening. For the heatmap representing the results of GSEA, the data were quantile normalized without performing signal + 1 and logarithm transformation. The Venny 2.1 online service was used to create a Venn diagram [69].

#### DEG analysis

DEGs were identified using the Bioconductor gene filter package in R [65]. To test whether there was a statistically significant difference between the groups, an independent *t*-test was applied to each gene of the pre-processed data. The statistical threshold for significance was a *p*-value < 0.05 and fold change >1.5 for DEGs derived from SOL OC/YC, SOL OE/OC, SOL YE/YC, GAS OC/YC, GAS OE/OC, and GAS YE/YC. The DEGs originating from SOL OC/GAS OC, SOL OE/GAS OE, SOL YC/GAS YC, and SOL YE/GAS YE were applied to *p*-value < 0.05 and fold change >2.0.

#### GO and KEGG pathway enrichment analysis

Functional annotation analysis was performed using the Database for Annotation, Visualization, and Integrated Discovery (DAVID) and KEGG. The criteria for statistically significant GO terms and KEGG pathways were FDR-corrected *p*-values < 0.05. To visualize the results of the enrichment analysis, the GOplot in R was utilized [70]. To facilitate interpretation, the z-score was calculated using the following formula:


$$\text{z}-\text{score}=\frac{\left(the\;number\;of\;upregulated\;genes)-(the\;number\;of\;downregulated\;genes\right)}{\sqrt{Total\;number\;of\;genes}}$$

#### GSEA

GSEA is a computational method that determines whether a priori defined set of genes shows statistically significant, concordant differences between two biological states. A gene set that satisfied *p*-value < 0.05 and FDR <0.25 was determined to be significant. “Mouse_Gene_Symbol_Remapping_ Human_Orthologs_MsigDB.v7.4.chip” was used as the chip platform. We conducted GSEA for SOL OC/YC, SOL OE/OC, SOL YE/YC, GAS OC/YC, GAS OE/OC, and GAS YE/YC.

## Supplementary Materials

Supplementary Tables

## References

[1] Zierath JR, Hawley JA. Skeletal muscle fiber type: influence on contractile and metabolic properties. PLoS Biol. 2004; 2:e348. 10.1371/journal.pbio.0020348 15486583 PMC521732

[2] Murgia M, Toniolo L, Nagaraj N, Ciciliot S, Vindigni V, Schiaffino S, Reggiani C, Mann M. Single Muscle Fiber Proteomics Reveals Fiber-Type-Specific Features of Human Muscle Aging. Cell Rep. 2017; 19:2396–409. 10.1016/j.celrep.2017.05.054 28614723

[3] Brunner F, Schmid A, Sheikhzadeh A, Nordin M, Yoon J, Frankel V. Effects of aging on Type II muscle fibers: a systematic review of the literature. J Aging Phys Act. 2007; 15:336–48. 10.1123/japa.15.3.336 17724398

[4] Grimby G. Muscle performance and structure in the elderly as studied cross-sectionally and longitudinally. J Gerontol A Biol Sci Med Sci. 1995; 50:17–22. 10.1093/gerona/50a.special_issue.17 7493212

[5] Gaitán JM, Moon HY, Stremlau M, Dubal DB, Cook DB, Okonkwo OC, van Praag H. Effects of Aerobic Exercise Training on Systemic Biomarkers and Cognition in Late Middle-Aged Adults at Risk for Alzheimer's Disease. Front Endocrinol (Lausanne). 2021; 12:660181. 10.3389/fendo.2021.660181 34093436 PMC8173166

[6] Yoon KJ, Zhang D, Kim SJ, Lee MC, Moon HY. Exercise-induced AMPK activation is involved in delay of skeletal muscle senescence. Biochem Biophys Res Commun. 2019; 512:604–10. 10.1016/j.bbrc.2019.03.086 30910357

[7] Hwang MH, Sim YJ. Vascular Endothelial Dysfunction and Exercise in Metabolic Syndrome Patients. Korean J Obes. 2015; 24:126–31. 10.7570/kjo.2015.24.3.126

[8] Miklosz A, Baranowski M, Lukaszuk B, Zabielski P, Chabowski A, Gorski J. Effect of acute exercise on mRNA and protein expression of main components of the lipolytic complex in different skeletal muscle types in the rat. J Physiol Pharmacol. 2019; 70:425–33. 10.26402/jpp.2019.3.09 31539888

[9] Shen F, Zhao Y, Ding W, Liu K, Ren X, Zhang Q, Yu J, Hu Y, Zuo H, Guo M, Jin L, Gong M, Wu W, et al. Autonomous climbing: An effective exercise mode with beneficial outcomes of aerobic exercise and resistance training. Life Sci. 2021; 265:118786. 10.1016/j.lfs.2020.118786 33221346

[10] Liu Y, Schlumberger A, Wirth K, Schmidtbleicher D, Steinacker JM. Different effects on human skeletal myosin heavy chain isoform expression: strength vs. combination training. J Appl Physiol (1985). 2003; 94:2282–8. 10.1152/japplphysiol.00830.2002 12736190

[11] Malisoux L, Francaux M, Nielens H, Theisen D. Stretch-shortening cycle exercises: an effective training paradigm to enhance power output of human single muscle fibers. J Appl Physiol (1985). 2006; 100:771–9. 10.1152/japplphysiol.01027.2005 16322375

[12] Andersen JL, Klitgaard H, Bangsbo J, Saltin B. Myosin heavy chain isoforms in single fibres from m. vastus lateralis of soccer players: effects of strength-training. Acta Physiol Scand. 1994; 150:21–6. 10.1111/j.1748-1716.1994.tb09655.x 8135120

[13] Luden N, Hayes E, Minchev K, Louis E, Raue U, Conley T, Trappe S. Skeletal muscle plasticity with marathon training in novice runners. Scand J Med Sci Sports. 2012; 22:662–70. 10.1111/j.1600-0838.2011.01305.x 21477203

[14] Jansson E, Sjödin B, Tesch P. Changes in muscle fibre type distribution in man after physical training. A sign of fibre type transformation? Acta Physiol Scand. 1978; 104:235–7. 10.1111/j.1748-1716.1978.tb06272.x 716974

[15] Augusto V, Padovani CR, Campos GER. Skeletal Muscle Fiber Types in C57BL6J Mice. J Morphol Sci. 2004; 21:89–94.

[16] Faitg J, Leduc-Gaudet JP, Reynaud O, Ferland G, Gaudreau P, Gouspillou G. Effects of Aging and Caloric Restriction on Fiber Type Composition, Mitochondrial Morphology and Dynamics in Rat Oxidative and Glycolytic Muscles. Front Physiol. 2019; 10:420. 10.3389/fphys.2019.00420 31114501 PMC6503296

[17] Fujiwara K, Asai H, Toyama H, Kunita K, Yaguchi C, Kiyota N, Tomita H, Jacobs JV. Changes in muscle thickness of gastrocnemius and soleus associated with age and sex. Aging Clin Exp Res. 2010; 22:24–30. 10.1007/BF03324811 19920407

[18] Lindwall C, Kanje M. Retrograde axonal transport of JNK signaling molecules influence injury induced nuclear changes in p-c-Jun and ATF3 in adult rat sensory neurons. Mol Cell Neurosci. 2005; 29:269–82. 10.1016/j.mcn.2005.03.002 15911351

[19] McKenzie MJ, Goldfarb AH, Kump DS. Gene response of the gastrocnemius and soleus muscles to an acute aerobic run in rats. J Sports Sci Med. 2011; 10:385–92. 24149887 PMC3761848

[20] Koçtürk S, Kayatekin BM, Resmi H, Açikgöz O, Kaynak C, Ozer E. The apoptotic response to strenuous exercise of the gastrocnemius and solues muscle fibers in rats. Eur J Appl Physiol. 2008; 102:515–24. 10.1007/s00421-007-0612-7 18030491

[21] Ogborn DI, Gardiner PF. Effects of exercise and muscle type on BDNF, NT-4/5, and TrKB expression in skeletal muscle. Muscle Nerve. 2010; 41:385–91. 10.1002/mus.21503 19813200

[22] Tarawan VM, Gunadi JW, Setiawan, Lesmana R, Goenawan H, Meilina DE, Sipayung JA, Wargasetia TL, Widowati W, Limyati Y, Supratman U. Alteration of Autophagy Gene Expression by Different Intensity of Exercise in Gastrocnemius and Soleus Muscles of Wistar Rats. J Sports Sci Med. 2019; 18:146–54. 30787662 PMC6370963

[23] Toth MJ, Callahan DM, Miller MS, Tourville TW, Hackett SB, Couch ME, Dittus K. Skeletal muscle fiber size and fiber type distribution in human cancer: Effects of weight loss and relationship to physical function. Clin Nutr. 2016; 35:1359–65. 10.1016/j.clnu.2016.02.016 27010836 PMC6411286

[24] Tanner CJ, Barakat HA, Dohm GL, Pories WJ, MacDonald KG, Cunningham PR, Swanson MS, Houmard JA. Muscle fiber type is associated with obesity and weight loss. Am J Physiol Endocrinol Metab. 2002; 282:E1191–6. 10.1152/ajpendo.00416.2001 12006347

[25] Oberbach A, Bossenz Y, Lehmann S, Niebauer J, Adams V, Paschke R, Schön MR, Blüher M, Punkt K. Altered fiber distribution and fiber-specific glycolytic and oxidative enzyme activity in skeletal muscle of patients with type 2 diabetes. Diabetes Care. 2006; 29:895–900. 10.2337/diacare.29.04.06.dc05-1854 16567834

[26] Kwak HB. Skeletal Muscle Mitochondria and Insulin Resistance: The Role of Exercise. Korean J Obes. 2015; 24:78–86. 10.7570/kjo.2015.24.2.78

[27] Hettige P, Tahir U, Nishikawa KC, Gage MJ. Comparative analysis of the transcriptomes of EDL, psoas, and soleus muscles from mice. BMC Genomics. 2020; 21:808. 10.1186/s12864-020-07225-2 33213377 PMC7678079

[28] Brandt SP. Microgenomics: gene expression analysis at the tissue-specific and single-cell levels. J Exp Bot. 2005; 56:495–505. 10.1093/jxb/eri066 15642711

[29] Uchitomi R, Hatazawa Y, Senoo N, Yoshioka K, Fujita M, Shimizu T, Miura S, Ono Y, Kamei Y. Metabolomic Analysis of Skeletal Muscle in Aged Mice. Sci Rep. 2019; 9:10425. 10.1038/s41598-019-46929-8 31320689 PMC6639307

[30] Strøm CC, Aplin M, Ploug T, Christoffersen TE, Langfort J, Viese M, Galbo H, Haunsø S, Sheikh SP. Expression profiling reveals differences in metabolic gene expression between exercise-induced cardiac effects and maladaptive cardiac hypertrophy. FEBS J. 2005; 272:2684–95. 10.1111/j.1742-4658.2005.04684.x 15943803

[31] Duan H, Chai J, Sheng Z, Yao Y, Yin H, Liang L, Shen C, Lin J. Effect of burn injury on apoptosis and expression of apoptosis-related genes/proteins in skeletal muscles of rats. Apoptosis. 2009; 14:52–65. 10.1007/s10495-008-0277-7 19009350

[32] Abreu-Vieira G, Fischer AW, Mattsson C, de Jong JM, Shabalina IG, Rydén M, Laurencikiene J, Arner P, Cannon B, Nedergaard J, Petrovic N. Cidea improves the metabolic profile through expansion of adipose tissue. Nat Commun. 2015; 6:7433. 10.1038/ncomms8433 26118629

[33] Zhou Z, Yon Toh S, Chen Z, Guo K, Ng CP, Ponniah S, Lin SC, Hong W, Li P. Cidea-deficient mice have lean phenotype and are resistant to obesity. Nat Genet. 2003; 35:49–56. 10.1038/ng1225 12910269

[34] Rogers NH, Landa A, Park S, Smith RG. Aging leads to a programmed loss of brown adipocytes in murine subcutaneous white adipose tissue. Aging Cell. 2012; 11:1074–83. 10.1111/acel.12010 23020201 PMC3839316

[35] Couppé C, Hansen P, Kongsgaard M, Kovanen V, Suetta C, Aagaard P, Kjaer M, Magnusson SP. Mechanical properties and collagen cross-linking of the patellar tendon in old and young men. J Appl Physiol (1985). 2009; 107:880–6. 10.1152/japplphysiol.00291.2009 19556458

[36] Haut RC, Lancaster RL, DeCamp CE. Mechanical properties of the canine patellar tendon: some correlations with age and the content of collagen. J Biomech. 1992; 25:163–73. 10.1016/0021-9290(92)90273-4 1733992

[37] Gachon F, Olela FF, Schaad O, Descombes P, Schibler U. The circadian PAR-domain basic leucine zipper transcription factors DBP, TEF, and HLF modulate basal and inducible xenobiotic detoxification. Cell Metab. 2006; 4:25–36. 10.1016/j.cmet.2006.04.015 16814730

[38] Woldt E, Sebti Y, Solt LA, Duhem C, Lancel S, Eeckhoute J, Hesselink MK, Paquet C, Delhaye S, Shin Y, Kamenecka TM, Schaart G, Lefebvre P, et al. Rev-erb-α modulates skeletal muscle oxidative capacity by regulating mitochondrial biogenesis and autophagy. Nat Med. 2013; 19:1039–46. 10.1038/nm.3213 23852339 PMC3737409

[39] Mayeuf-Louchart A, Thorel Q, Delhaye S, Beauchamp J, Duhem C, Danckaert A, Lancel S, Pourcet B, Woldt E, Boulinguiez A, Ferri L, Zecchin M, Staels B, et al. Rev-erb-α regulates atrophy-related genes to control skeletal muscle mass. Sci Rep. 2017; 7:14383. 10.1038/s41598-017-14596-2 29085009 PMC5662766

[40] Yu JM, Wu X, Gimble JM, Guan X, Freitas MA, Bunnell BA. Age-related changes in mesenchymal stem cells derived from rhesus macaque bone marrow. Aging Cell. 2011; 10:66–79. 10.1111/j.1474-9726.2010.00646.x 20969724 PMC4339051

[41] Harper JM, Wilkinson JE, Miller RA. Macrophage migration inhibitory factor-knockout mice are long lived and respond to caloric restriction. FASEB J. 2010; 24:2436–42. 10.1096/fj.09-152223 20219983 PMC2887269

[42] Aoki N, Kimura S, Xing Z. Role of DAP12 in innate and adaptive immune responses. Curr Pharm Des. 2003; 9:7–10. 10.2174/1381612033392503 12570670

[43] Rovina RL, da Rocha AL, Marafon BB, Pauli JR, de Moura LP, Cintra DE, Ropelle ER, da Silva ASR. One Bout of Aerobic Exercise Can Enhance the Expression of *Nr1d1* in Oxidative Skeletal Muscle Samples. Front Physiol. 2021; 12:626096. 10.3389/fphys.2021.626096 33597895 PMC7882602

[44] Chen D, Mauvais-Jarvis F, Bluher M, Fisher SJ, Jozsi A, Goodyear LJ, Ueki K, Kahn CR. p50alpha/p55alpha phosphoinositide 3-kinase knockout mice exhibit enhanced insulin sensitivity. Mol Cell Biol. 2004; 24:320–9. 10.1128/MCB.24.1.320-329.2004 14673165 PMC303335

[45] Pataky MW, Yu CS, Nie Y, Arias EB, Singh M, Mendias CL, Ploutz-Snyder RJ, Cartee GD. Skeletal muscle fiber type-selective effects of acute exercise on insulin-stimulated glucose uptake in insulin-resistant, high-fat-fed rats. Am J Physiol Endocrinol Metab. 2019; 316:E695–706. 10.1152/ajpendo.00482.2018 30753114 PMC6580167

[46] Pingitore P, Romeo S. The role of PNPLA3 in health and disease. Biochim Biophys Acta Mol Cell Biol Lipids. 2019; 1864:900–6. 10.1016/j.bbalip.2018.06.018 29935383

[47] Ajami M, Atashi A, Kaviani S, Kiani J, Soleimani M. Generation of an in vitro model of β-thalassemia using the CRISPR/Cas9 genome editing system. J Cell Biochem. 2020; 121:1420–30. 10.1002/jcb.29377 31596028

[48] Tikkanen HO, Näveri HK, Härkönen MH. Alteration of regulatory enzyme activities in fast-twitch and slow-twitch muscles and muscle fibres in low-intensity endurance-trained rats. Eur J Appl Physiol Occup Physiol. 1995; 70:281–7. 10.1007/BF00865023 7649137

[49] Shimizu N, Yoshikawa N, Ito N, Maruyama T, Suzuki Y, Takeda S, Nakae J, Tagata Y, Nishitani S, Takehana K, Sano M, Fukuda K, Suematsu M, et al. Crosstalk between glucocorticoid receptor and nutritional sensor mTOR in skeletal muscle. Cell Metab. 2011; 13:170–82. 10.1016/j.cmet.2011.01.001 21284984

[50] Rice KM, Manne ND, Gadde MK, Paturi S, Arvapalli R, Blough E. Differential regulation of apoptosis in slow and fast twitch muscles of aged female F344BN rats. Age (Dordr). 2015; 37:30. 10.1007/s11357-015-9767-z 25813803 PMC4375133

[51] Leeuwenburgh C, Gurley CM, Strotman BA, Dupont-Versteegden EE. Age-related differences in apoptosis with disuse atrophy in soleus muscle. Am J Physiol Regul Integr Comp Physiol. 2005; 288:R1288–96. 10.1152/ajpregu.00576.2004 15650125

[52] Marzetti E, Groban L, Wohlgemuth SE, Lees HA, Lin M, Jobe H, Giovannini S, Leeuwenburgh C, Carter CS. Effects of short-term GH supplementation and treadmill exercise training on physical performance and skeletal muscle apoptosis in old rats. Am J Physiol Regul Integr Comp Physiol. 2008; 294:R558–67. 10.1152/ajpregu.00620.2007 18003794

[53] Alway SE, Degens H, Krishnamurthy G, Chaudhrai A. Denervation stimulates apoptosis but not Id2 expression in hindlimb muscles of aged rats. J Gerontol A Biol Sci Med Sci. 2003; 58:687–97. 10.1093/gerona/58.8.b687 12902526

[54] Dirks A, Leeuwenburgh C. Apoptosis in skeletal muscle with aging. Am J Physiol Regul Integr Comp Physiol. 2002; 282:R519–27. 10.1152/ajpregu.00458.2001 11792662

[55] Dirks AJ, Leeuwenburgh C. Aging and lifelong calorie restriction result in adaptations of skeletal muscle apoptosis repressor, apoptosis-inducing factor, X-linked inhibitor of apoptosis, caspase-3, and caspase-12. Free Radic Biol Med. 2004; 36:27–39. 10.1016/j.freeradbiomed.2003.10.003 14732288

[56] Rice KM, Blough ER. Sarcopenia-related apoptosis is regulated differently in fast- and slow-twitch muscles of the aging F344/N x BN rat model. Mech Ageing Dev. 2006; 127:670–9. 10.1016/j.mad.2006.03.005 16678239

[57] Azad M, Khaledi N, Hedayati M, Karbalaie M. Apoptotic response to acute and chronic exercises in rat skeletal muscle: Eccentric & sprint interval. Life Sci. 2021; 270:119002. 10.1016/j.lfs.2020.119002 33417954

[58] Margaritelis NV, Theodorou AA, Chatzinikolaou PN, Kyparos A, Nikolaidis MG, Paschalis V. Eccentric exercise per se does not affect muscle damage biomarkers: early and late phase adaptations. Eur J Appl Physiol. 2021; 121:549–59. 10.1007/s00421-020-04528-w 33156414

[59] Nourshahi M, Rostami S, Khodagholi F. Effect of Eight Weeks Sprint Interval Training on Myogenin Rate in Aged Rats Skeletal Muscle Tissue Maryam. Med J Tabriz Uni Med Sci Heal Serv. 2017; 39:76–82.

[60] Borselli C, Storrie H, Benesch-Lee F, Shvartsman D, Cezar C, Lichtman JW, Vandenburgh HH, Mooney DJ. Functional muscle regeneration with combined delivery of angiogenesis and myogenesis factors. Proc Natl Acad Sci U S A. 2010; 107:3287–92. 10.1073/pnas.0903875106 19966309 PMC2840452

[61] Shefer G, Rauner G, Yablonka-Reuveni Z, Benayahu D. Reduced satellite cell numbers and myogenic capacity in aging can be alleviated by endurance exercise. PLoS One. 2010; 5:e13307. 10.1371/journal.pone.0013307 20967266 PMC2953499

[62] Moritani T, Oddsson L, Thorstensson A. Activation patterns of the soleus and gastrocnemius muscles during different motor tasks. J Electromyogr Kinesiol. 1991; 1:81–8. 10.1016/1050-6411(91)90001-L 20870497

[63] Lee M, Cho HS, Yoon KJ, Lee W, Moon HY. Exercise-induced changes of gene expression in the cerebellum of aged mice. Biochem Biophys Res Commun. 2020; 521:952–6. 10.1016/j.bbrc.2019.11.024 31718796

[64] Bolstad B. preprocessCore: A collection of pre-processing functions. R package version 1.57.1, 2022. https://github.com/bmbolstad/preprocessCore.

[65] Gentleman AR, Carey V, Huber W, Hahne F. genefilter: methods for filtering genes from microarray experiments. 2013. https://www.yumpu.com/en/document/read/26666751/package-genefilter-bioconductor.

[66] Holland SM. Principal Component Analysis (PCA). 2019. https://strata.uga.edu/software/pdf/pcaTutorial.pdf.

[67] Vu VQ. ggbiplot: A ggplot2 based biplot. R package version 0.55, 755. 2011. https://rdocumentation.org/packages/ggbiplot/versions/0.55.

[68] Gu Z, Eils R, Schlesner M. Complex heatmaps reveal patterns and correlations in multidimensional genomic data. Bioinformatics. 2016; 32:2847–9. 10.1093/bioinformatics/btw313 27207943

[69] Oliveros JC. VENNY. An interactive tool for comparing lists with Venn Diagrams. 2007. http://bioinfogp.cnb.csic.es/tools/venny/index.html.

[70] Walter W, Sánchez-Cabo F, Ricote M. GOplot: an R package for visually combining expression data with functional analysis. Bioinformatics. 2015; 31:2912–4. 10.1093/bioinformatics/btv300 25964631

