# Supplementary material for: Muscle fiber type-dependence effect of exercise on genomic networks in aged mice models
Source: Aging (Albany NY). 2022 Apr 19;14(8):3337–64. doi: 10.18632/aging.204024 (PMC9085230; doi:10.18632/aging.204024)
Supplement: Supplementary Tables [file aging-14-204024-s001.pdf]

SUPPLEMENTARY TABLES

Supplementary Table 1. The number of DEGs by aging and exercise effects in SOL and GAS.

Group	Up-regulated	Down-regulated	Total
**SOL**			
OC/YC	90	106	196
YE/YC	11	20	31
OE/OC	10	10	20
**GAS**			
OC/YC	71	48	119
YE/YC	7	6	13
OE/OC	6	7	13

Supplementary Table 2. The number of DEGs between SOL and GAS in YC, YE, OC and OE.

Group	Up-regulated	Down-regulated	Total
**YC**			
SOL/GAS	539	521	1060
**YE**			
SOL/GAS	459	432	891
**OC**			
SOL/GAS	542	510	1052
**OE**			
SOL/GAS	540	520	1060

Supplementary Table 3. The significant enriched GO terms of DEGs between SOL YC and GAS YC.

ID	Term	Adjusted *p*.value
GO:0006936	Muscle contraction	0
GO:0006631	Fatty acid metabolic process	0
GO:0006629	Lipid metabolic process	0.0001
GO:0006635	Fatty acid beta-oxidation	0.0001
GO:0030239	Myofibril assembly	0.0001
GO:0008152	Metabolic process	0.0001
GO:0060048	Cardiac muscle contraction	0.0002
GO:0010880	Regulation of release of sequestered calcium ion into cytosol by sarcoplasmic reticulum	0.0002
GO:0007519	Skeletal muscle tissue development	0.0002
GO:0006937	Regulation of muscle contraction	0.0002
GO:0006096	Glycolytic process	0.0002
GO:0055114	Oxidation-reduction process	0.0003
GO:0003009	Skeletal muscle contraction	0.0006
GO:0006094	Gluconeogenesis	0.0006
GO:0014883	Transition between fast and slow fiber	0.0006
GO:0045214	Sarcomere organization	0.0028
GO:0033539	Fatty acid beta-oxidation using acyl-CoA dehydrogenase	0.0043
GO:0006941	Striated muscle contraction	0.0058
GO:0055010	Ventricular cardiac muscle tissue morphogenesis	0.0081
GO:0051289	Protein homotetramerization	0.0108
GO:0055008	Cardiac muscle tissue morphogenesis	0.0117

GO:0086004	Regulation of cardiac muscle cell contraction	0.0117
GO:0002026	Regulation of the force of heart contraction	0.0129
GO:0045822	Negative regulation of heart contraction	0.0150
GO:0001676	Long-chain fatty acid metabolic process	0.0214
GO:0006470	Protein dephosphorylation	0.0214
GO:0007155	Cell adhesion	0.0219
GO:0030049	Muscle filament sliding	0.0224
GO:0006950	Response to stress	0.0294
GO:0060316	Positive regulation of ryanodine-sensitive calcium-release channel activity	0.0402

Supplementary Table 4. The significant enriched KEGG pathway of DEGs between SOL YC and GAS YC.

ID	Term	Adjusted *p*.value
mmu01130	Biosynthesis of antibiotics	0
mmu01200	Carbon metabolism	0
mmu00071	Fatty acid degradation	0
mmu00010	Glycolysis / Gluconeogenesis	0
mmu04261	Adrenergic signaling in cardiomyocytes	0
mmu01212	Fatty acid metabolism	0
mmu01100	Metabolic pathways	0
mmu04260	Cardiac muscle contraction	0.0001
mmu05410	Hypertrophic cardiomyopathy (HCM)	0.0001
mmu00280	Valine, leucine and isoleucine degradation	0.0001
mmu05414	Dilated cardiomyopathy	0.0001
mmu01230	Biosynthesis of amino acids	0.0001
mmu00620	Pyruvate metabolism	0.0004
mmu00410	beta-Alanine metabolism	0.0006
mmu00640	Propanoate metabolism	0.0007
mmu04922	Glucagon signaling pathway	0.0008
mmu03320	PPAR signaling pathway	0.0008
mmu00330	Arginine and proline metabolism	0.0023
mmu04530	Tight junction	0.0027
mmu00062	Fatty acid elongation	0.0032
mmu04921	Oxytocin signaling pathway	0.0032
mmu04810	Regulation of actin cytoskeleton	0.0032
mmu00650	Butanoate metabolism	0.0036
mmu05205	Proteoglycans in cancer	0.0036
mmu04974	Protein digestion and absorption	0.0056
mmu04964	Proximal tubule bicarbonate reclamation	0.0062
mmu00270	Cysteine and methionine metabolism	0.0077
mmu04971	Gastric acid secretion	0.0095
mmu04510	Focal adhesion	0.0095
mmu04010	MAPK signaling pathway	0.0110
mmu00051	Fructose and mannose metabolism	0.0117
mmu04970	Salivary secretion	0.0148
mmu04022	cGMP-PKG signaling pathway	0.0148
mmu04919	Thyroid hormone signaling pathway	0.0162
mmu00380	Tryptophan metabolism	0.0173
mmu04020	Calcium signaling pathway	0.0173

mmu00030	Pentose phosphate pathway	0.0244
mmu04910	Insulin signaling pathway	0.0383
mmu04066	HIF-1 signaling pathway	0.0396

Supplementary Table 5. The significant enriched GO terms of DEGs between SOL OC and GAS OC.

ID	Term	Adjusted *p*.value
GO:0006936	Muscle contraction	0
GO:0006631	Fatty acid metabolic process	0
GO:0010880	Regulation of release of sequestered calcium ion into cytosol by sarcoplasmic reticulum	0
GO:0060048	Cardiac muscle contraction	0
GO:0007519	Skeletal muscle tissue development	0.0001
GO:0030239	Myofibril assembly	0.0001
GO:0006629	Lipid metabolic process	0.0001
GO:0008152	Metabolic process	0.0001
GO:0006937	Regulation of muscle contraction	0.0002
GO:0045214	Sarcomere organization	0.0004
GO:0003009	Skeletal muscle contraction	0.0007
GO:0014883	Transition between fast and slow fiber	0.0007
GO:0086004	Regulation of cardiac muscle cell contraction	0.0013
GO:0006096	Glycolytic process	0.0014
GO:0002026	Regulation of the force of heart contraction	0.0018
GO:0060316	Positive regulation of ryanodine-sensitive calcium-release channel activity	0.0028
GO:0006094	Gluconeogenesis	0.0043
GO:0006941	Striated muscle contraction	0.0053
GO:0006635	Fatty acid beta-oxidation	0.0069
GO:0002027	Regulation of heart rate	0.0072
GO:0051289	Protein homotetramerization	0.0085
GO:0045822	Negative regulation of heart contraction	0.0149
GO:0055114	Oxidation-reduction process	0.0210
GO:0030049	Muscle filament sliding	0.0235
GO:0001666	Response to hypoxia	0.0235
GO:0006883	Cellular sodium ion homeostasis	0.0365
GO:0055010	Ventricular cardiac muscle tissue morphogenesis	0.0381
GO:0048739	Cardiac muscle fiber development	0.0400
GO:0019886	Antigen processing and presentation of exogenous peptide antigen via MHC class II	0.0434

Supplementary Table 6. The significant enriched KEGG pathway of DEGs between SOL OC and GAS OC.

ID	Term	Adjusted *p*.value
mmu01130	Biosynthesis of antibiotics	0
mmu01200	Carbon metabolism	0
mmu04922	Glucagon signaling pathway	0
mmu00010	Glycolysis / Gluconeogenesis	0
mmu01212	Fatty acid metabolism	0.0003
mmu04261	Adrenergic signaling in cardiomyocytes	0.0003
mmu00620	Pyruvate metabolism	0.0005
mmu04910	Insulin signaling pathway	0.0006

mmu00071	Fatty acid degradation	0.0006
mmu04260	Cardiac muscle contraction	0.0006
mmu05410	Hypertrophic cardiomyopathy (HCM)	0.0007
mmu00640	Propanoate metabolism	0.0007
mmu04970	Salivary secretion	0.0018
mmu04530	Tight junction	0.0023
mmu04921	Oxytocin signaling pathway	0.0027
mmu03320	PPAR signaling pathway	0.0083
mmu05414	Dilated cardiomyopathy	0.0106
mmu04971	Gastric acid secretion	0.0106
mmu04810	Regulation of actin cytoskeleton	0.0123
mmu00051	Fructose and mannose metabolism	0.0133
mmu05205	Proteoglycans in cancer	0.0133
mmu01230	Biosynthesis of amino acids	0.0133
mmu04022	cGMP-PKG signaling pathway	0.0133
mmu04020	Calcium signaling pathway	0.0162
mmu01100	Metabolic pathways	0.0257
mmu05152	Tuberculosis	0.0276
mmu04964	Proximal tubule bicarbonate reclamation	0.0324
mmu04919	Thyroid hormone signaling pathway	0.0365
mmu04145	Phagosome	0.0421
